# Perturbations in L-serine metabolism regulate protein quality control through the sensor of the retrograde response pathway *RTG2* in *Saccharomyces cerevisiae*

**DOI:** 10.1016/j.jbc.2025.110329

**Published:** 2025-05-31

**Authors:** Kanika Saxena, Rebecca Andersson, Per O. Widlund, Sakda Khoomrung, Sarah Hanzén, Jens Nielsen, Navinder Kumar, Mikael Molin, Thomas Nyström

**Affiliations:** 1 Department of Immunology and Microbiology, Institute for Biomedicine, Gothenburg University, Göteborg, Sweden; 2 Translational Science and Experimental Medicine, Early Research and Development, Respiratory and Immunology, Biopharmaceuticals R&D, AstraZeneca AB, Mölndal, Sweden; 3 Stem Cell Aging Group, Program for Clinical Translation of Regenerative Medicine in Catalonia (P-CMR[C]), Institut d’Investigació Biomèdica de Bellvitge (IDIBELL), L’Hospitalet de Llobregat, Barcelona, Spain; 4 Systems and Synthetic Biology, Department of Life Sciences, Chalmers University of Technology, Gothenburg, Sweden; 5 Siriraj Center of Research Excellence in Metabolomics and Systems Biology (SiCORE-MSB), Faculty of Medicine Siriraj Hospital, Mahidol University, Bangkok, Thailand; 6 Siriraj Metabolomics and Phenomics Center, Faculty of Medicine Siriraj Hospital, Mahidol University, Bangkok, Thailand; 7 Department of Biochemistry, Faculty of Medicine Siriraj Hospital Mahidol University, Bangkok, Thailand; 8 Clinical Strategy and Innovation, Cochlear Bone Anchored Solutions AB, Mölnlycke, Sweden

**Keywords:** aggregation, aging, proteostasis, mitochondria, serine, retrograde response, replicative aging

## Abstract

Cellular protein homeostasis relies on a complex network of protein synthesis, folding, sub-cellular localization, and degradation to sustain a functional proteome. Since most of these processes are energy-driven, proteostasis is inescapably afflicted by cellular metabolism. Proteostasis collapse and metabolic imbalance are both linked to aging and age-associated disorders, yet they have traditionally been studied as separate phenomena in the context of aging. In this study, we indicate that reduced proteostasis capacity is a result of a metabolic imbalance associated with age. We observed increased accumulation of L-serine and L-threonine in replicative old cells of *Saccharomyces cerevisiae*, indicating an imbalance in amino acid metabolism with replicative aging. Replicating this metabolic imbalance in young cells through deletion of serine-dependent transcriptional activator, *CHA4*, resulted in increased aggregation of endogenous proteins along with misfolding-prone proteins Guk1-7ts-GFP and Luciferase-GFP in both young and old cells. Aggregate formation in the *cha4Δ* strain required a functional sensor of mitochondrial dysfunction and an activator of the retrograde signaling gene, *RTG2*. *CHA4* and *RTG2* exhibited genetic interaction and together regulated mitochondrial metabolism, replicative lifespan, and aggregate formation in young cells, connecting metabolic regulation with proteostasis and aging. Constitutive activation of retrograde signaling through overexpression of *RTG2* or deletion of *MKS-1*, a negative regulator of Rtg1-Rtg3 nuclear translocation, resulted in faster resolution of aggregates upon heat shock through *RTG3* and was found to be independent of molecular chaperone upregulation.

Cellular protein homeostasis (proteostasis) is achieved through the concerted efforts of protein quality control (PQC) components comprising protein synthesis, folding, degradation, trafficking, and spatial sequestration mechanisms (1, 2, 3, 4). Inability to mount stress responses, protein aggregation, and inefficient degradation of cellular proteins are examples of proteostasis decline occurring during aging and are also hallmarks of age-associated diseases (5, 6). Proteostasis restoration following proteotoxic insults requires degradation of misfolded and aggregated proteins together with the *de novo* synthesis of nascent polypeptides linking these two processes with the cellular pool of amino acids (2). Since both protein synthesis and degradation are ATP-dependent processes, these are inescapably affected by cellular energy metabolism. At a cellular and organismal level, limitations in energy and nutrient availability activate nutrient responsive genes and stress response pathways that act to restore metabolic homeostasis in the cell (7, 8, 9, 10). These nutrient-regulated stress response pathways are intricately coupled with protein homeostasis (proteostasis) pathways. Conversely, altered proteostasis capacity can lead to perturbations in energy homeostasis and the development of a variety of metabolic diseases including diabetes (11, 12). In addition, some specific metabolites have the capacity to directly influence folding of mutant polypeptides both *in vivo* and *in vitro* and are therefore referred to as chemical chaperones (13, 14, 15). Thus, it is important to understand the functional link between metabolic regulation and protein quality control, as both have been largely considered separately in the context of aging.

Altered mitochondrial function and morphology is one key driver of aging and age-associated disease pathologies (16). Retrograde signaling, which responds to mitochondrial dysfunction in yeast and other organisms, activates the expression of a cascade of genes that leads to mitochondrial biogenesis and metabolic changes that ultimately restore mitochondrial function and prolong lifespan (10, 17, 18). The Rtg2 protein acts as a sensor of mitochondrial dysfunction and activates retrograde signaling. During replicative aging of yeast cells, mitochondrial dysfunction is due to the disruption of proton-dependent neutral amino acid storage in vacuoles (19). A recent study identified that mitochondria-derived compartments (MDCs) are mitochondrial structures with distinct lumen enriched in outer and intermembrane proteins Tom70 and oxaloacetate carrier Oac1, respectively and depleted intermembrane protein Tim50 and matrix protein acetolactate synthase Ilv2 (20, 21). These compartments are formed upon elevated amino acid levels in the cytosol and regulate metabolic adaptation under such conditions by sequestering SLC25A, an outer mitochondrial membrane nutrient carrier, and its associated importer Tom70 (21). Furthermore, aggregation-prone cytosolic proteins are imported into mitochondria for degradation during heat shock, and blocking this translocation delays degradation of the aggregated proteins (22). Thus, mitochondria are an important hub connecting metabolism and proteostasis.

Here, we report on a link between proteostasis imbalance and metabolic perturbation in *Saccharomyces cerevisiae*. We show that replicative old cells of yeast accumulate L-serine and L-threonine, suggesting an altered amino acid metabolism in old cells. This metabolic perturbation of L-serine and L-threonine metabolism in old cells could be mimicked by deleting the *CHA4* gene, encoding a serine-dependent transcriptional activator, and resulted in an increased aggregation of misfolding-prone proteins GUK1-7ts-GFP, Luciferase-GFP, and endogenous proteins in both young and old cells. We also found that in the absence of *CHA4*, *RTG2* function is required to maintain mitochondrial metabolism, replicative lifespan, and aggregate formation in young cells both under normal conditions and during heat shock. Constitutive activation of retrograde signaling through overexpression of *RTG2* or deletion of *MKS1 (negative regulator of Rtg1-Rtg3 nuclear translocation)* resulted in faster resolution of aggregates upon heat shock through the downstream effector *RTG3*. This effect did not require upregulation of molecular chaperone biosynthesis.

## Results

### Identification of metabolites that show altered accumulation in old cells

**Figure 1:**
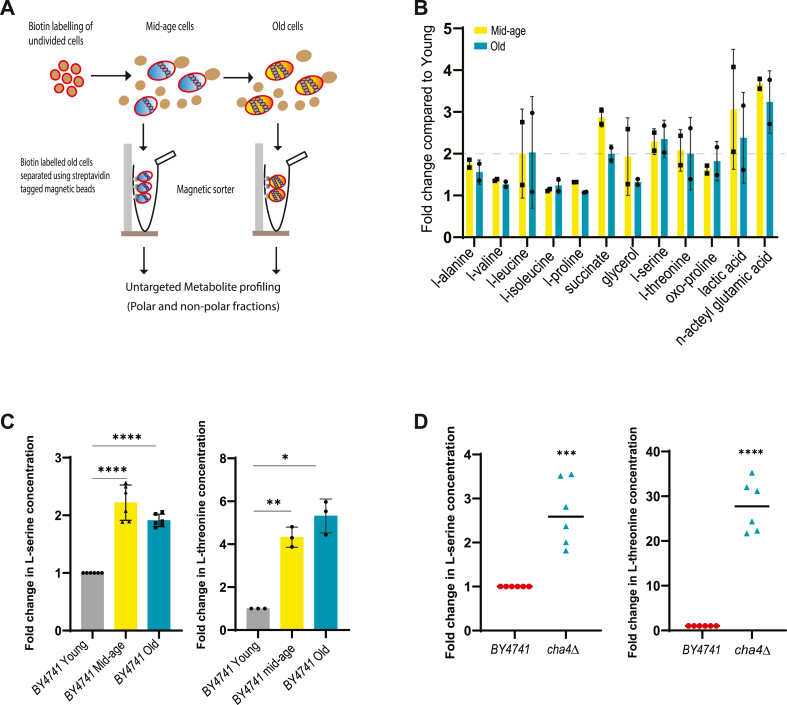
**L-serine and L-threonine accumulates in BY4741 old cells and in *cha4*Δ**. *A*, schematic of old cell isolation and metabolite profiling through GC-MS ISQ-LT or GC-FID. *B*, representative graph depicting metabolites with two-fold increase in concentration in mid-age and old cells, identified through untargeted metabolite profiling using GC-MS (two replicates out of three plotted, N = 2). Peak area of the metabolite was normalized with the internal standard 10 mM anthranilic acid (0.1 M HCL), log_2_ values for peak area per weight is plotted. *C*, fold change increase in L-serine (N = 6) and L-threonine concentrations (N = 3) in BY4741 mid-age & old cells w.r.t young cells is plotted on Y-axis, concentrations were determined using amino acid standard, and analysis performed using GC-FID; parametric paired *t* test (∗∗∗∗) *p* < 0.0001, (∗∗) *p*-value = 0.007, (∗) *p*-value = 0.011. *D*, fold change increase in L-serine (N = 6) and L-threonine (N = 6) concentrations in young cells of *cha4*D w.r.t BY4741; concentrations were determined using amino acid standard, and analysis was performed using GC-FID; parametric paired *t* test (∗∗∗∗) *p* < 0.0001, (∗∗∗) *p*-value = 0.0004.

To identify metabolites that alter expression with replicative age, we examined a wild-type strain of *S*. *cerevisiae*, BY4741, and isolated cells at three different stages of their replicative lifespan (RLS); young (log-phase cells), mid-age (11 bud scars), and old (17 bud scars) cells and performed untargeted metabolomics for both polar and non-polar fractions using GC-MS/MS ISQ-LT analysis (Fig. 1*A*). The peaks in GC-MS/MS spectra were identified using a reference standard (23), and the peak areas were normalized against the internal standard 10 mM anthranilic acid (in 0.1 M HCL). In the initial run, we detected 37 peaks corresponding to both identified and unknown metabolites. Among the 27 identified metabolites, we observed 18 amino acids (except cysteine and methionine) and TCA cycle intermediates as well as glycerol (Fig. S1*A*). Intracellular accumulation for most of these metabolites remained unchanged with age (Figs. 1*B* & S1*A*). Metabolites whose levels were elevated twofold or more in mid- and old-age cells included lactic acid, succinate, L-serine, L-threonine, and N-acetyl glutamic acid (Figs. 1*B*). Since only a few distinct metabolites show more than a two-fold change in their intracellular levels during mid- and old-age, old cells do not appear to undergo global changes in their cellular metabolism (Figs. 1*B* & S1, *B* and *C*). Out of the metabolites changing in levels, we chose the L-serine metabolic network comprising L-serine and L-threonine as our target for further investigation. L-serine has been linked to protein quality control as it accelerates *in vitro* refolding of two model proteins, DM-MBP and GFP (14) and its cellular uptake increases during proteotoxic thermal stress (24, 25) in yeast. Thus, we speculated that accumulation of L-serine in old cells could have consequences on protein folding and aggregation, and we thus confirmed that L-serine levels, indeed, go up by twofold in BY4741 mid-age and old cells compared with young cells, using the targeted approach based on GC-FID analysis (Fig. S1*C*). We also measured the concentration of L-serine in both young and old cells of the Mca1OE strain, which exhibits an extended replicative lifespan through mechanisms that are independent of metabolic regulation (26). Mca1OE strain showed identical levels of L-serine accumulation with age compared to BY4741 (Fig. S1*D*), suggesting that accumulation of L-serine with age is a general phenomenon.

### Altered L-serine metabolism undermines protein quality control

To test whether dysregulated L-serine metabolism affects cellular protein quality control, we selected *cha4*Δ deletion mutant known to be affected by serine metabolism and elucidated to what extent, if any, this mutation affected protein quality control. The Cha4 is a transcriptional activator that regulates intracellular L-serine levels by repressing Ser3 (encoding the 3-phosphoglycerate dehydrogenase and alpha-ketoglutarate reductase enzyme that catalyzes the first step in the biosynthesis of L-serine) and activating L-serine catabolism by the Cha1 (catabolic L-serine/L-threonine deaminase enzyme) (27). A *CHA4* deletion has previously been shown to result in twofold higher levels of intracellular L-serine in synthetic complete (SC) medium (28). In our targeted analysis of *cha4*Δ cells, we observed 2 to 3 fold higher intracellular L-serine and 24-fold higher L-threonine levels w.r.t BY4741 in the YPD medium, which partially matches with previously published data (28) (Fig. 1*D*), and mimics the situation in replicative old cells (Fig. 1, *B* and *C*).

**Figure 2:**
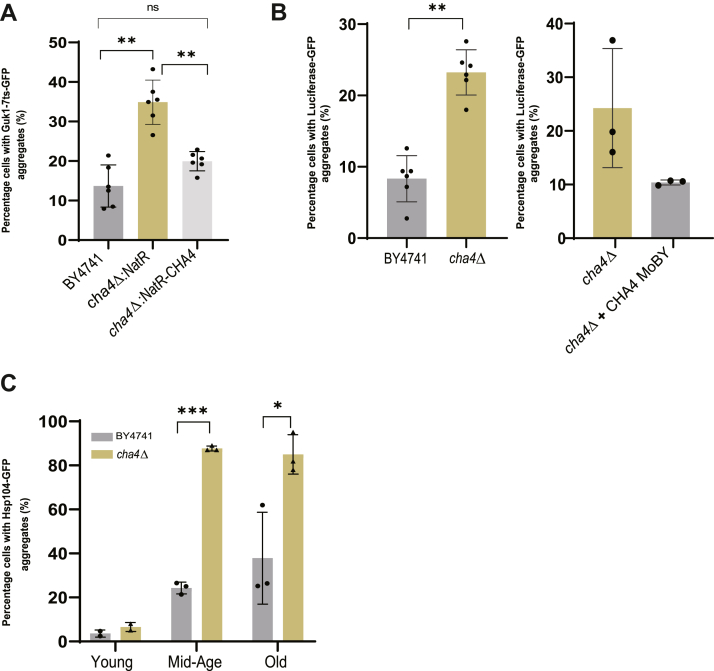
***CHA4* deletion impairs proteostasis in young and old cells**. *A*, *cha4*Δ expressing Guk1-7ts-GFP-HIS was complemented with Nat resistance gene alone (control) or with NatR-CHA4, percentage cells with aggregates were quantified (N = 6); one-way ANOVA *p*-value=<0.0001, parametric paired *t* test (∗∗) *p*-value = 0.005 for BY4741 vs *cha4Δ*; *p*-value = 0.003 for cha4 vs *cha4Δ-NatR-CHA4*. *B*, BY4741 and *cha4*Δ were transformed with Luciferase-GFP-HIS (N = 6), *cha4*Δ was transformed with MoBY control plasmid and MoBY-CHA4 plasmid for complementation (N = 3), percentage of cells with aggregates was quantified; parametric paired *t* test (∗∗) *p*-value = 0.003. *C*, percentage of cells with aggregates of BY4741 and *cha4*Δ carrying genomic insertion of Hsp104-GFP-HIS were quantified (N = 3); 2-way ANOVA row X coloumn factor *p*-value = 0.0013, row-factor *p*-value = 0.0026, column-factor *p*-value<0.0001, subject *p*-value = 0.045; parametric paired *t* test (∗∗∗) *p*-value = 0.0001, (∗) *p*-value = 0.02. Old cells were isolated as described in the methods and the percentage of cells with aggregates was quantified using imageJ software.

Next, we produced the misfolding reporter protein Guk1-7(ts)-GFP to further determine the status of protein homeostasis in *cha4Δ* strain. *GUK1* encodes yeast guanylate kinase. Guk1-7ts-GFP is a heat labile protein and aggregates at restrictive temperatures (29, 30). Since, temperature sensitive mutant proteins have previously been shown to aggregate with age at permissive temperatures in *Caenorhabditis elegans* (5), we used Guk1-7(ts)-GFP as a sensor to report on the cellular folding environment of *cha4Δ* (31). The wild-type copy of the gene *GUK1* is present in all the strains used for analysis ensuring that its function is not perturbed (32). Interestingly, when Guk1-7ts-GFP was expressed in the *cha4*Δ genetic background, we observed a significantly higher percentage of young cells with aggregates (35%) compared to BY4741 at a normal growth temperature 30^o^C (Figs. 2*A* & S2*A*). To confirm that the observed difference is due to the gene deletion and not secondary mutations elsewhere in the genome, we introduced the *CHA4* gene back at the URA locus in the *cha4*Δ mutant using the Nourseothricin N-acetyl transferase resistance marker (*NatR*). This resulted in complementation of the Guk1-7ts-GFP aggregate phenotype (Figs. 2*A* & S2*A*). We also confirmed that the increased levels of aggregate formation in *cha4*Δ is not due to the increased levels of protein expression, using western blot analysis (Fig. S3*G*). This suggests a possible link between dysregulated L-serine metabolism and compromised protein quality control.

To confirm this finding, we used an unrelated misfolding reporter protein Luciferase-GFP that does not have a function in the yeast cells. The Luciferase-GFP protein, when produced in BY4741, forms aggregates in cells in the diauxic phase of growth. The *cha4*Δ strain displayed a twofold higher percentage of cells with Luciferase-GFP aggregates compared with BY4741 during the diauxic phase of growth (Figs. 2*B* & S2*B*). This phenotype was also complemented when we introduced the *CHA4* gene back on a MoBY plasmid (33) (Figs. 2*B* & S2*B*). This, together with the data on Guk1-7ts-GFP aggregate formation, suggests that dysregulated L-serine metabolism results in compromised cellular protein quality control and promotes protein aggregation both during the log phase and diauxic phase of growth.

To further study the effect of perturbation of L-serine metabolism on the aggregation of endogenous proteins, we tagged the disaggregase Hsp104 expressed through its endogenous promoter with GFP in the *cha4Δ* strain. Hsp104 co-localizes with cellular aggregates, and its GFP-tagging allows visualization and quantification of aggregates of endogenous aggregated proteins (34, 35, 36, 37). We isolated young, mid-age, and old cells of BY4741 and *cha4Δ* strains, and counted cells with Hsp104-GFP foci. We observed that both BY4741 and the *cha4Δ* strain displayed a diffuse Hsp104-GFP signal during their young cell stage (Fig. 2*C* & S2*C*). However, during the mid-age *cha4Δ* had a fourfold higher percentage of cells with aggregates compared to BY4741 (Fig. 2*C*). Taken together, these findings suggest that the intracellular accumulation of metabolites resulting from L-serine metabolic network perturbation can directly affect protein homeostasis and aggregate formation both in young and in old cells.

L-serine accumulation can increase metabolic flux in the endoplasmic reticulum (ER) and mitochondria. In the ER, serine is crucial for sphingolipid biosynthesis, initiated by serine palmitoyl transferase (SPT complex), which condenses serine with palmitoyl-CoA. We used myriocin to inhibit the SPT complex. Myriocin treatment led to no change in aggregate formation in *cha4*Δ (Fig. S2*D*), indicating that serine's flux through the sphingolipid biosynthetic pathway does not disrupt proteostasis in *cha4*Δ. Since serine and glycine are interconverted by cytosolic and mitochondrial serine hydroxymethyl transferase (*SHM1/2*), feeding substrates into the one-carbon cycle, we measured Shm1/2 protein levels *via* western blot and found no significant difference between the BY4741 and *cha4Δ* mutant (Fig. S3*H*), suggesting that the proteostasis imbalance in *cha4Δ* is likely not due to serine’s flux through one-carbon regulon.

### CHA4 and RTG2 genetically interact to regulate protein quality control and aging

While we were conducting this study, a research article was published by Muller *et al*. (28), where they performed amino acid profiling for 4913 gene deletion strains of yeast. They observed L-serine and L-threonine as two of the most concentration-stable amino acids across the gene deletions tested. Therefore, we examined their raw data to identify gene deletions causing deviations in intracellular L-serine concentration. Interestingly, their data showed that a *rtg2*Δ strain displayed a markedly diminished concentration of intracellular serine (0.499 mM) compared to the wild type and most of the other gene deletion strains, where the concentrations were found to be more robust with an average cellular concentration of 2.3 mM (28). The *RTG2* gene acts as a sensor of mitochondrial dysfunction and activates the retrograde response, resulting in nuclear gene expression that initiates major changes in cellular metabolism (17, 18, 38, 39). The association of the *RTG2* gene with mitochondrial function and cellular metabolism makes it an interesting candidate for investigating whether intracellular serine accumulation could have deleterious effects on PQC through mitochondrial dysfunction.

**Figure 3:**
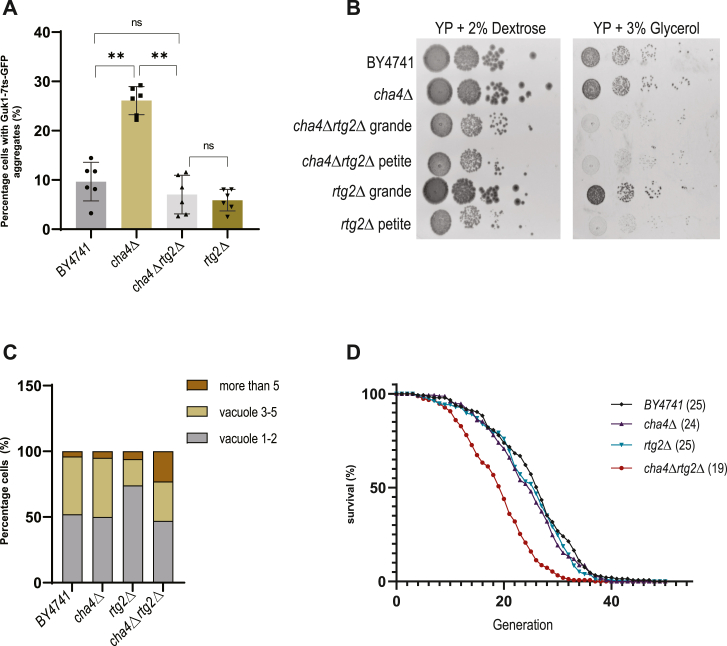
**Genetic interaction between *CHA4* and *RTG2***. *A*, percentage of cells with Guk1-7ts-GFP aggregates (%) (N = 6), cells were grown at 30^o^C; one-way ANOVA *p*-value<0.0001; parametric paired *t* test *p*-value = 0.004 BY4741 vs *cha4*Δ, *p*-value = 0.009 *cha4Δ* vs *cha4Δrtg2Δ*. *B*, spot assay of tenfold serially diluted logarithmic phase cultures on YP plates containing 2% glucose or 3% glycerol. *C*, percentage cells with different numbers of vacuoles were counted (100 cells), vacuoles were stained with FM4-64 days). *D*, replicative lifespan of BY4741, *cha4*Δ, *cha4Δrtg2Δ* and *rtg2Δ*, all four strains expressed Guk1-7ts-GFP; mean replicative lifespan of each strain is indicated in parentheses. Mann Whitney test performed (∗) *p*-value = 0.03 *BY4741*(27) vs *cha4Δrtg2Δ* (21). Percentage cells with aggregates were quantified using imageJ software.

We deleted *RTG2* in the *cha4Δ* strain expressing Guk1-7ts-GFP and found that removing *RTG2* suppressed aggregate formation in the *cha4*Δ strain to the levels comparable to the wild type cells (Fig. 3*A*). We confirmed using western blot analysis that this suppression was not due to the differences in protein expression levels between *cha4Δ* & *cha4Δrtg2*Δ (Fig. S3*G*). We also measured the activity of proteasome by observing the rate of degradation of its substrate CTL^∗^ (40) (Fig. S3*F*). We observed reduced proteasome activity in *cha4Δrtg2*Δ, with a half-life of 90 min, compared to *cha4Δ*'s 49 min, this difference does not account for the observed reduction in aggregate formation within *cha4Δrtg2*Δ (Fig. S3*F*). The suppression of aggregation in *cha4Δrtg2*Δ was, however, accompanied by a markedly reduced growth rate, suggesting a genetic epistatic interaction between *CHA4* and *RTG2* (Fig. 3*B* & S3*B*). To eliminate the possibility that a reduction in the percentage of cells with aggregates could result from reduced translation due to a slower growth rate, cells were grown in the presence of a cycloheximide concentration, which inhibited the growth rate of *cha4Δ* comparable to *cha4Δrtg2Δ*. Reducing translation with cycloheximide chase did not mimic the reduction of aggregation seen by deleting *RTG2* (Fig. S3*C*). We also found that the *rtg2Δ* strain displayed two colony morphologies: petite and grande as reported previously in the literature (17). There was no difference in the percentage of cells with aggregates between *rtg2*Δ petite and grande colonies at 30^o^C, which resembled BY4741 (Fig. S3*A*). Further, only petite colonies were deficient in a mitochondrial metabolic function of utilizing glycerol as an alternate source of carbon (Fig. 3*B*). We observed that the *cha4Δrtg*2Δ double mutant also displayed petite and grande colonies but that, in this strain, cells of both colony morphologies were deficient in the utilization of glycerol as a carbon source (Fig. 3*B*). Thus, removing *RTG2* caused a complete block in glycerol utilization in cells lacking *CHA4*. Furthermore, the double mutant, *cha4Δrtg2Δ*, like *rtg2Δ*, was also unable to utilize acetate as a sole carbon source, whereas *cha4Δ* did not exhibit this deficiency (Fig. S3*E*). However, no significant difference in Shm1/2 protein levels was observed between *cha4Δ* and *cha4Δrtg2Δ* when measured by Western blot (Fig. S3*H*), suggesting no likely defect in one carbon regulon in the absence of *RTG2*. Additionally, mitoSOX red staining indicated that *cha4Δrtg2Δ* maintained a similar mitochondrial redox potential to that of BY4741 (Fig. S3*J*). These observations suggest that *RTG2* is required to maintain specific mitochondrial metabolic functions, such as, growth on respiratory substrates like glycerol, and acetate in *cha4Δ* mutant.

In addition, double mutant *cha4Δrtg*2Δ cells displayed an approximately 4- to 5-fold increase in the percentage of cells with fragmented vacuoles (Fig. 3*C*). Thus, as the double mutant shows reduced aggregate formation accompanied with the signs of reduced fitness and cellular stress, *e*.*g*., vacuolar fragmentation and defects in specific mitochondrial metabolic functions (Figs. 3*B*, S3, *E* and *C*), aggregate formation seems to be a protective measure ensured by the activity of Rtg2 in *cha4*Δ cells.

Since L-serine metabolic network is interlinked with other amino acids like L-threonine, glycine, cysteine, and aspartate, perturbation in this metabolic network could alter the activity of TOR (Target of Rapamycin) signaling pathway and thus could regulate proteostasis through general stress response (41, 42, 43). To test this, we treated *cha4*Δ and *cha4Δrtg2Δ* with rapamycin and did not observe any difference in the percentage of cells with aggregates compared to the untreated control (Fig. S3*D*). Intracellular serine levels regulate glutathione biosynthesis, impacting redox homeostasis (44). We evaluated the susceptibility of *cha4*Δ and *cha4Δrtg*2Δ to oxidative stress using a spot assay with 1mM H_2_O_2_. No growth defects were observed in either strain under these conditions (Fig. S3*I*).

Next, we evaluated the effect of the *RTG2* deletion in the *cha4*Δ background on replicative lifespan. The wild type BY4741 strain displayed a mean RLS of 27 divisions; both the *cha4*Δ and the *rtg2*Δ strains displayed a similar lifespan (24 and 25 divisions respectively), whereas the *cha4Δrtg*2Δ had a significantly shorter lifespan with mean RLS of 21 divisions, strengthening the notion that *RTG2* has a protective function in the absence of *CHA4* gene activity (Fig. 3*D*).

### The role of Rtg2 in proteostasis in cells with aberrant serine metabolism is independent of canonical retrograde and SAGA/SLIK signaling

**Figure 4:**
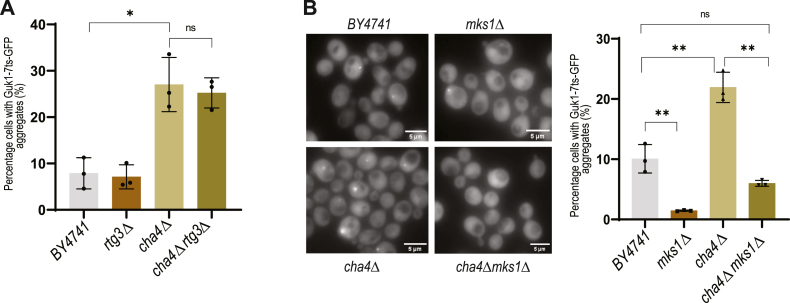
***RTG2* and *MKS1* regulate proteostasis in *cha4*Δ through non-canonical interaction**. *A*, percentage cells with Guk1-7ts-GFP aggregates (%) (N = 3), cells were grown at 30 °C; one-way ANOVA *p*-value = 0.0003; parametric paired *t* test (∗) *p*-value = 0.014 BY4741 vs *cha4*Δ. *B*, percentage cells with Guk1-7ts-GFP aggregates (%) (N = 3), one-way ANOVA *p*-value<0.0001; cells were grown at 30 °C; parametric paired *t* test (∗∗) *p*-value = 0.008 BY4741 vs *cha4*Δ, (∗∗) *p*-value = 0.005 *cha4Δ* vs *cha4Δmks1Δ*, (∗∗) *p*-value = 0.002 *BY4741* vs *mks1Δ*; cells were imaged using fluorescence microscope and images were analysed by imageJ software.

Mitochondrial dysfunction in *S*. *cerevisiae* activates retrograde signaling resulting in metabolic adaptations and the activation of a stress response (18). The myriad of genes that are activated ensures the reestablishment of cellular homeostasis and increases replicative lifespan of yeast (18, 45, 46, 47). Activation of retrograde signaling requires entry of the heterodimeric transcription factor Rtg1-Rtg3 into the nucleus (39). This translocation requires partial dephosphorylation of Rtg3 which is presumed to be caused by the phosphatase activity of Rtg2 (39). Also, the negative regulator of retrograde signaling, MAP kinase substrate 1 (Mks1), forms a complex with either Bmh1 or Bmh2 and causes hyperphosphorylation of Rtg3 sequestering it in the cytosol (48). Rtg2 binds Mks1 and prevents its binding to Bmh1/Bmh2, activating Rtg1-Rtg3 translocation, acting as a positive regulator of retrograde signaling (48, 49). As discussed in the previous section (Fig. 3), Rtg2 function seems to be important for aggregate formation and cellular quality control in *cha4*Δ mutant cells. To test if activation of retrograde signaling through translocation of Rtg1-Rtg3 into the nucleus in *cha4*Δ cells could be involved in this process, we deleted *RTG3* in the *cha4*Δ background and measured its effect on Guk1-7ts-GFP aggregates. In contrast to our expectations, the *RTG3* deletion did not have any effect on Guk1-7ts-GFP aggregate formation in *cha4*Δ cells, suggesting that the processes upstream of Rtg1-Rtg3 nuclear translocation could be involved (Fig. 4*A*). In another approach, we constitutively activated retrograde signaling in the *cha4Δ* strain by deleting the negative regulator Mks1. Interestingly, *mks1*Δ itself had significantly reduced percentage of cells with aggregates compared to BY4741 (Fig. 4*B* & S4*A*) and *cha4*Δ*mks1*Δ was comparable to BY4741(Fig. 4*B* & S4*A*). Previous studies have shown that Mks1p and Rtg2p are present in a complex which excludes Rtg3p and Mks1p directly binds Rtg2p *via* its C-terminal domain (48, 50), suggesting that Rtg2p-Mks1p may act together to regulate aggregate formation in *cha4Δ* strain through previously unidentified pathway that does not require Rtg3p function. Taken together, these results suggest that regulators of retrograde signaling *RTG2* and *MKS1* affect aggregate formation and proteostasis in cells lacking *CHA4*; however, the exact interplay between these genes in regulating aggregate formation remains elusive. Also, the network of genes that are activated by Rtg1-Rtg3 transcription complex seems to be dispensable for proteostasis in *cha4Δ* cells.

Rtg2 has recently been shown to be a component of the SAGA-like transcriptional co-activator complex known as SLIK (51). The Spt-Ada-Gcn5-Acetyl transferase complex (SAGA) activates stress responsive genes by modulating post-transcriptional histone modifications. Rtg2 replaces Spt8 in the SAGA complex to form SLIK. To test if the aggregate formation in *cha4*Δ cells is regulated by the activation of the SLIK or SAGA stress responses, we deleted the gene *SPT7*, which encodes a protein required for the stability of both the multimeric protein complexes. *SPT7* deletion in the *cha4Δ* had no effect on Guk1-7ts-GFP aggregate formation (Fig. S4*B*). We also deleted *GCN5*, which constitutes the acetyl transferase activity of both SAGA and SLIK, *cha4*Δ*gcn5*Δ had a slightly higher percentage of cells with aggregates compared to *cha4*Δ (Fig. S4*C*). These results suggest that in cells with aberrant serine metabolism, the Rtg2 regulator encompasses hitherto unknown functions unrelated to its role in retrograde and SAGA/SLIK signaling.

### Rtg2-dependent activation of retrograde signaling accelerates the clearance of heat-induced aggregates

**Figure 5:**
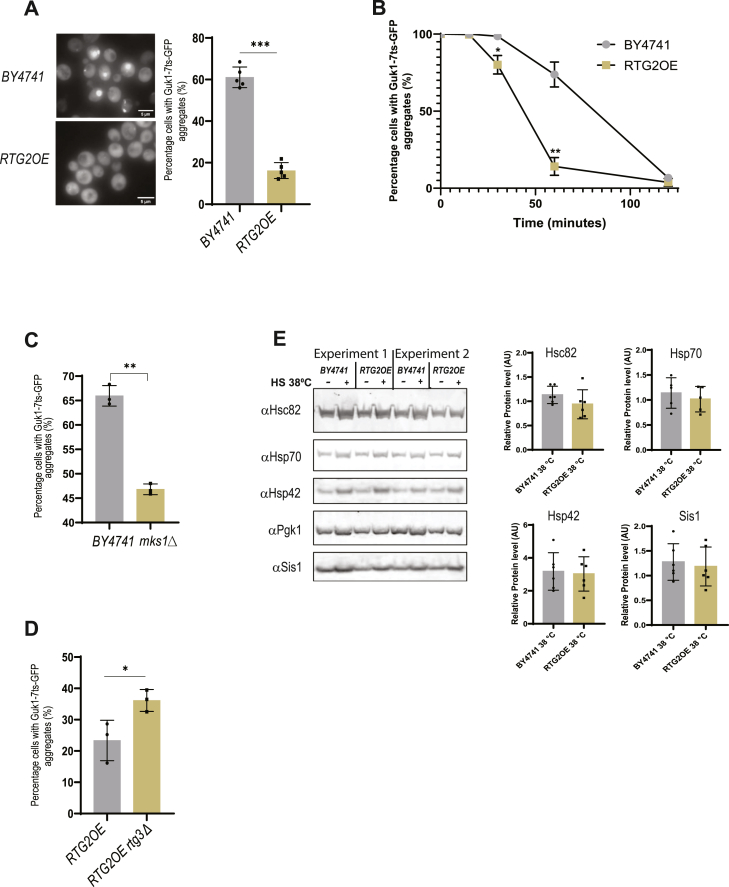
***RTG2* overexpression regulates aggregate formation upon heat shock *via* retrograde signaling**. *A*, BY4741 and *RTG2*OE strain expressing Guk1-7ts-GFP were heat shocked at 38 °C for 90 min, Percentage cells with Guk1-7ts-GFP aggregates (%) (N = 5), parametric paired *t* test (∗∗∗) *p*-value = 0.0004. *B*, aggregate clearance rate of RTG2OE compared with BY4741 (N = 3), log phase cells grown at 30 °C were heat shocked at 42 °C, percentage cells with Guk1-7ts-GFP aggregates were quantified. One-way Anova (*p* < 0.0001); paired *t* test, 30 min *p*-value = 0.029 & 60 min *p*-value = 0.004. *C*, BY4741 and *mks1Δ* strain expressing Guk1-7ts-GFP were heat shocked at 38 °C for 90 min, Percentage cells with Guk1-7ts-GFP aggregates (%) (N = 3), parametric paired *t* test (∗∗) *p*-value = 0.008 *D*, *RTG2*OE, & *RTG2*OE *rtg3Δ* strains expressing Guk1-7ts-GFP were heat shocked at 38 °C for 90 min, Percentage cells with Guk1-7ts-GFP aggregates (%) (N = 3), parametric paired *t* test (∗) *p*-value = 0.019 *RTG2OE* vs *RTG2*OE *rtg3Δ*. *E*, indicated strains expressing Guk1-7ts-GFP were heat shocked at 38 °C for 30 min and chaperone levels were measured using Western blot. Images were quantified using ImageJ software and the levels of chaperones in each lane were normalized against *PGK1* housekeeping gene.

Since an *RTG2* deletion regulates aggregate formation of a thermolabile protein, Guk1-7ts-GFP in *cha4*Δ (Fig. 3*A*), we tested the effect of a constitutive high expression of *RTG2* on Guk1-7ts-GFP aggregate formation upon heat shock. *RTG2* was overexpressed by replacing its endogenous promoter with the *GPD* promoter in BY4741 cells carrying genomic Guk1-7ts-GFP, and the cells were heat shocked at 38^o^C for 90 min. Interestingly, we observed a drastic reduction in the percentage of cells with aggregates in the *RTG2* overproducer (*RTG2*OE) compared with BY4741 (Figs. 5*A* & S5*A*). To validate that this effect is not just limited to the Guk1-7ts-GFP protein, we overproduced *RTG2* in a BY4741 wild-type strain carrying Pro3-1ts-GFP, another thermolabile protein that misfolds and aggregates at higher temperatures (29). We observed the same effect of *RTG2* overproduction on Pro3-1ts-GFP aggregation as with Guk1-7ts-GFP (Fig. S5*B*). *RTG2*OE also showed a significantly reduced percentage of cells with Guk1-7ts-GFP aggregates in synthetic complete medium at 30 °C (Fig. S5*C*). The lower percentage of cells with aggregates upon heat shock in the *RTG2* overproducer strain could be due to two reasons: the inability to form aggregates upon heat shock or a faster disaggregation, or both. To investigate these possibilities, we followed cells at various time points after initiating a heat shock at 38 °C & 42 °C and found that the *RTG2* overproducer resolved the aggregates much faster than the wild-type cells both at 38 °C and 42 °C (Figs. 5*B*, S5, *D* and *E*).

In an alternate strategy to activate the retrograde response constitutively, we deleted *MKS1* in BY4741 *GUK1-7ts-GFP* background. As with *RTG2* overproduction, the *MKS1* deletion caused a reduction in the percentage of cells with aggregates (45%) upon a heat shock compared with BY4741 (67%) (Fig. 5*C*). We found that the deletion of *RTG3* in the *RTG2*OE *GUK1-7ts-GFP* background resulted in a slight increase in the percentage of cells with aggregation, suggesting involvement of retrograde signaling in regulating aggregate formation upon heat shock (Fig. 5*D*).

Since, upon heat shock, HSF-1 is activated, which in turn activates the synthesis of molecular chaperones, we analyzed the levels of different molecular chaperones (Sis1, Hsp42, Hsp70, and Hsc82) that are normally induced upon heat shock, in the *RTG2*OE strain. Interestingly, the levels of molecular chaperones were either induced to the same level as the wild type upon heat shock (Sis1 and Hsp42) or remained uninduced (Hsp70 and Hsc82) (Figs. 5*E* &S5*F*). We also analyzed whether the faster resolution of aggregates in the *RTG2OE* could be due to enhanced activity of HSF-1. We expressed a plasmid carrying an HSF-1 responsive promoter upstream of the luciferase gene in the *BY4741*, *RTG2*OE, and *rtg2Δ* strains and analyzed activation of HSF-1 at 30 ^o^C (52). We observed that the *RTG2*OE strain displayed a reduced activity of HSF-1 compared to BY4741 (Fig. S5*G*). This suggests that in the *RTG2*OE strain, activation of retrograde signaling facilitates faster disaggregation of heat-induced aggregates by a mechanism other than the induction of molecular chaperones. Although RTG2OE strain was efficient in clearing aggregates upon heat shock, it showed shorter lifespan (Fig. S5*H*), reduced growth rate (Fig. S5*I*), and was unable to utilize glycerol and acetate as a carbon source (Figs. S5*I*, and S3*E*).

## Discussion

Proteostasis collapse and metabolic imbalance are both hallmarks of aging and age-associated diseases; however, little is known about their interdependence, as the two processes have been largely considered separately in the context of aging (12). Here, we show that replicative old cells of *S*. *cerevisiae* accumulate intracellular L-serine and L-threonine and genetic manipulations leading to perturbation of the L-serine metabolic network have direct consequences on cellular protein quality control (Figures 1 and 2). Interestingly, L-serine metabolism is dysregulated in cancer cells (53). Extracellular supply and *de novo* synthesis of L-serine is sufficient to promote cancer cell proliferation (54) and restricting L-serine and L-glycine in the diet can promote tumor regression (55). Since age is a risk factor for developing cancer, our findings provide an insight into the plausible implication of age associated dysregulation of L-serine metabolism on cellular health and protein quality control.

Cellular metabolite uptake, accumulation, and synthesis are fine-tuned with extracellular nutrient availability. *CHA4* gene deletion resulted in a metabolic insult, making yeast cells impaired in regulating intracellular L-serine and L-threonine concentrations both in nutrient-rich and diauxic phases. This caused misfolding and aggregation of aggregation-prone proteins both in young and old cells (Fig. 2 & S2), suggesting that cells with an altered metabolism are susceptible to protein misfolding and aggregation under conditions of nutrient stress and during aging.

We show for the first time that there is genetic interaction between *RTG2* and *CHA4* (Fig. 3). *RTG2* is required both for aggregate formation and for the regulation of specific mitochondrial metabolism in the *cha4*Δ background (Figs. 3*B*, & S3*E*). Serine flux through one carbon regulon is important for mitochondrial translation initiation (56, 57). Also, increased serine levels impose mitochondrial metabolic stress by generating high levels of 2-aminoacrylate leading to loss of mitochondrial DNA (58). A recent study in yeast reported the formation of MDCs in response to amino acid elevation. MDCs sequester nutrient carriers (SLC25A) and import receptor Tom70 and facilitate amino acid catabolism in the mitochondria (21). Thus, it seems plausible that in conditions of amino acid excess in the cytosol in strains such as *cha4*Δ, cells respond by activating mechanisms involved in regulating mitochondrial metabolism and health (Fig. 3). Involvement of Rtg2 in regulating aggregate formation under such conditions strengthens the idea of metabolic regulation of proteostasis. However, the canonical retrograde response pathway seems to be dispensable for aggregate formation in *cha4*Δ and Rtg2 and Mks1 seems to interact *via* an unidentified mechanism which requires further investigation (Fig. 4). Since Rtg2 and Mks1 proteins interact physically (48), it is possible that they may have regulatory functions other than activating retrograde response signaling.

In this work, we present findings suggesting the involvement of retrograde signaling in accelerating aggregate clearance upon heat shock and that this effect is mediated by effector *RTG3* (Figs. 5*B* & S5, *D* and *E*). Interestingly, the faster resolution of aggregates in Rtg2 overproducer strain is not the result of increased molecular chaperone activity (Fig. 5*E*). However, in the *cha4*Δ strain, *RTG2* function in aggregate formation is independent of *RTG3* and SLIK (Fig. 4). Since Cha4 is also a transcription factor, changes in gene expression pattern in its absence may modify Rtg2 interactions and functions. These findings point towards a mechanism of protein quality control by Rtg2 which requires in-depth investigation.

Based on our findings, we propose that replicative old cells of *S*. *cerevisiae* accumulate L-serine, which overwhelms the mitochondria for its degradation and clearance. Since mitochondrial functions are already compromised in aging cells this presents as an additional challenge to cope with. *RTG2* and *CHA4* genes interact to regulate mitochondrial metabolism and aggregate formation throughout the replicative lifespan of the yeast cells and provide an example of metabolic regulation of protein quality control in the context of the replicative lifespan of yeast.

## Experimental procedures

### Strains and statistics

Yeast strains used in the study are derivatives of BY4741 with genotype MATa *his3Δ*1 l*eu2Δ*0 *met15*Δ0 *ura3*Δ0. Single-gene deletion strains were obtained from the yeast knockout collection (59). Yeast strains were grown in yeast extract/peptone (YP) + 2% dextrose (YPD) for all experiments. Yeast strains expressing Luciferase-GFP (pCA837) (60) were grown in synthetic complete medium containing yeast nitrogen base with ammonium sulphate, synthetic complete His drop-out, and 2% dextrose. Statistical analysis was performed using GraphPad prism© 8 (version 8.4.3). One-way or two-way ANOVA was performed to determine the significance of the datasets, combined with a parametric paired *t* test to determine individual *p*-values between groups.

### Old cell isolation

Overnight cultures were diluted in YP containing 2% dextrose (YPD) and incubated at 30^o^C for 5 hours until OD_600_=0.5. Logarithmic phase culture was harvested at 4 °C, washed in ice-cold phosphate-buffered saline (PBS), and labeled with 5 mg of sulfo-NHS-LC biotin (Pierce). Cells were then washed with 50 ml of PBS pH 8 to remove excess biotin and resuspended in YPD for overnight growth. Biotin-labeled cells grown overnight were harvested at 4 °C and washed with PBS. Cells were incubated with 250 μl of streptavidin-conjugated paramagnetic beads (5 mg/ml, Pierce) for 2 hours at 4 °C. Cells bound to streptavidin magnetic beads were isolated using a magnetic sorter. For imaging, live cells were used, and for mass spectrometry analysis, cells were weighed and quenched using methanol at −80 °C temperature. Cells were harvested to remove methanol and stored at −80 °C until further analysis. Half of the cells bound to streptavidin beads were inoculated in YPD for further round of divisions. The same procedure was followed as above to isolate old cells bound to streptavidin beads.

### Metabolite profiling by GC-MS

The samples were prepared as described in detail by (23). Briefly, 50 μl of anthranilic acid (10 mM in 0.1 M HCl) was added to the cell pellet and used as an internal standard. Cellular metabolites were extracted in 1:1 mixture of 50% methanol and chloroform. The samples were vigorously shaken at −20 °C for 45 min and centrifuged at 2900g for 5 min. The two phases were collected and pooled in fresh tubes. The mixture was kept for drying in a vacuum concentrator overnight at room temperature. The samples were then resuspended in 1:1 mixture of 50% aqueous methanol and chloroform and transferred to silanized GC vials following brief vortexing. Samples were derivatized in 400 μl of acetonitrile and 100 μl of t-BDMS solution. 10 mM anthranilic acid (in 0.1 M HCl) was aliquoted into three vials and was derivatized with the same protocol. The samples were incubated at 80 °C overnight and analyzed by GC-MS. The metabolite derivatives were separated by Gas chromatography using Focus GC ISQ single quadrupole GC-MS (Thermo Fisher Scientific). 1 μl of the sample was injected in the splitless mode, helium gas as a carrier with flow rate at 1 ml/min as previously described (23). Identification of the unknown metabolites was done by comparing their retention time and mass spectrum profiles with reference standards. N-acetyl glutamic acid and L-lactic acid peaks were identified using National Institute of Standards and Technology (NIST) library. Data processing was performed using the Quan browser function in Xcalibur software version 2.2 (Thermo Fisher Scientific).

### Gas chromatography-flame ionization detection (GC-FID) analysis

Metabolites were extracted in 2.5 mL each of pre-cooled 50% aqueous methanol and chloroform. Samples were then kept for shaking at 2000 rpm at −20 °C for 45 min and centrifuged at −20 °C at 2900g for 5 min. Only the methanol fraction was collected and used for kit-based sample preparation, the easy-fast amino acid sample testing kit (EZ:faast) from Phenomenex, according to the manufacturer's instructions. L-serine and L-threonine peaks were identified, and their concentration was determined by retention time and peak area comparison with the amino acid standard provided with the kit. Xcalibur software from Thermo Fisher Scientific was used for analysis.

### Fluorescence microscopy and image analysis

Live cells were imaged using a fluorescence microscope, Zeiss Axio Observer.Z1 inverted microscope with apotome and AxioCam 506 camera, Plan-apochromat 100x/1.40 oil DIC M27 objective. For all experiments, 7 to 9 z-stacks were captured. Images were analyzed by ImageJ software, and the percentage of cells with aggregates was counted using the cell counter plug-in. Statistical analysis was performed using GraphPad Prism 8.4.3 (GraphPad Software LLC).

### Lifespan analysis

Lifespan analysis was performed as described in (61). Single cells from log-phase culture were placed on YPD agar plates using a micromanipulator and allowed to bud once. The mother cells were removed, and the newly formed daughter cells were considered for counting the total number of divisions. The experiment was performed twice with 70 to 80 cells from each strain. Statistical analysis was performed using GraphPad Prism 8.4.3 (GraphPad Software LLC).

### Spot assay

Cells grown at 30 °C to mid-exponential phase were serially diluted tenfold (1:10^1^-1:10^5^) and plated on YP plates containing 2% dextrose or YP plates containing 3% glycerol. Plates were incubated at 30 °C for 2 days to allow maximum growth.

### Aggregate clearance assay

Indicated strains were grown in YPD at 30 °C to mid-exponential phase, transferred to 42 °C for 30 min in a water bath, followed by recovery at 30 °C. Samples were taken at indicated times and fixed in 3.7% formaldehyde for 30 min at room temperature. Cells were washed 2x with PBS and stored at 4 °C before imaging. The percentage of cells with aggregates was quantified using FIJI/Image J software.

### Heat shock for Western blot

Cells were grown to mid-exponential phase at 30 °C in minimal media, after which 1 O.D. of cells was taken as a control sample. The remainder of each culture was shifted to 38 °C in a pre-heated shaking water bath, and a sample of 1 O.D. of cells was taken at the indicated times for protein extraction.

### Protein extraction for Western blot

One O.D. of cells was collected and lysed with 0.2 M NaOH for 20 min of ice. The proteins were pelleted by centrifugation at 13,000 RPM for 1 min at 4 °C and the protein pellet was resuspended in 50 μl of sample buffer (1X Laemmli/8 M Urea/2.5% β-mercaptoethanol). Prior to loading on a gel, the protein suspension was incubated at 70 °C for 10 min.

### SDS-Page and western blot

SDS-PAGE was performed using 4 to 12% Bis-Tris gels (Invitrogen NuPAGE, Thermo Scientific) in a MOPS buffer (Invitrogen NuPAGE, Thermo Scientific) according to the manufacturer’s instruction. The gels were blotted onto an Immobilion-P PVDF membrane, 0.45 μm pore size, (MERCK Millipore) in a Tris-glycine-methanol transfer buffer overnight. The following antibodies were used for protein detection: 1:5000 monoclonal mouse anti-Hsp70 (Abcam), 1:15,000 monoclonal mouse anti-Pgk1 (Thermo Fisher Scientific), 1: 20,000 rabbit anti-Hsp42 (a gift from Professor Johannes Buchner), 1:10,000 polyclonal rabbit anti-Hsc82 (Abcam) and 1:10,000 rabbit anti-Sis1. The primary antibodies were detected using 1:20,000 goat anti-mouse or rabbit IgG (H + L) IRDye 680LT or 800CW (mouse 680LT: LI-COR Biosciences, mouse 800CW: LI-COR Biosciences, rabbit 680LT: LI-COR Biosciences, rabbit 800CW: LI-COR Biosciences), the blots were scanned using the Odyssey Near Infra-red imaging system (LI-COR) and the images were analyzed using ImageJ. The levels of *PGK1*, a house -keeping gene were used to normalize the levels of indicated chaperone in each lane.

## Data availability

Data is available on request knksaxena@gmail.com, thomas.nystrom@cmb.gu.se.

## Supporting information

This article contains supporting information (40, 52, 59, 62, 63).

## Conflict of interest

The authors declare that they have no conflicts of interest with the contents of this article.

## Supporting information

Figure S2

Figure S3 part 1

Figure S3 part 1

## References

[1] Labbadia J., Morimoto R.I. (2015). The biology of proteostasis in aging and disease. Annu. Rev. Biochem..

[2] Jayaraj G.G., Hipp M.S., Hartl F.U. (2020). Functional modules of the proteostasis network. Cold. Spring. Harb. Perspect. Biol..

[3] Hipp M.S., Kasturi P., Hartl F.U. (2019). The proteostasis network and its decline in ageing. Nat. Rev. Mol. Cell. Biol..

[4] Hill S.M., Hanzén S., Nyström T. (2017). Restricted access: spatial sequestration of damaged proteins during stress and aging. EMBO Rep..

[5] Ben-Zvi A., Miller E.A., Morimoto R.I. (2009). Collapse of proteostasis represents an early molecular event in Caenorhabditis elegans aging. Proc. Natl. Acad. Sci. U. S. A..

[6] López-Otín C., Blasco M.A., Partridge L., Serrano M., Kroemer G. (2023). Hallmarks of aging: an expanding universe. Cell.

[7] Conrad M., Schothorst J., Kankipati H.N., Van Zeebroeck G., Rubio-Texeira M., Thevelein J.M. (2014). Nutrient sensing and signaling in the yeast Saccharomyces cerevisiae. FEMS Microbiol. Rev..

[8] Kapahi P., Zid B. (2004). TOR pathway: linking nutrient sensing to life span. Sci. Aging. Knowledge. Environ..

[9] Barbieri M., Bonafè M., Franceschi C., Paolisso G. (2003). Insulin/IGF-I-signaling pathway: an evolutionarily conserved mechanism of longevity from yeast to humans. Am. J. Physiol. Endocrinol. Metab..

[10] Jazwinski S.M., Kriete A. (2012). The yeast retrograde response as a model of intracellular signaling of mitochondrial dysfunction. Front. Physiol..

[11] Morimoto R.I., Cuervo A.M. (2014). Proteostasis and the aging proteome in health and disease. J. Gerontol. Ser. A.

[12] Ottens F., Franz A., Hoppe T. (2021). Build-UPS and break-downs: metabolism impacts on proteostasis and aging. Cell. Death. Differ..

[13] Bandyopadhyay A., Saxena K., Kasturia N., Dalal V., Bhatt N., Rajkumar A. (2012). Chemical chaperones assist intracellular folding to buffer mutational variations. Nat. Chem. Biol..

[14] Dandage R., Bandyopadhyay A., Jayaraj G.G., Saxena K., Dalal V., Das A. (2015). Classification of chemical chaperones based on their effect on protein folding landscapes. ACS. Chem. Biol..

[15] Verma K., Saxena K., Donaka R., Chaphalkar A., Rai M.K., Shukla A. (2020). Distinct metabolic states of a cell guide alternate fates of mutational buffering through altered proteostasis. Nat. Commun..

[16] Amorim J.A., Coppotelli G., Rolo A.P., Palmeira C.M., Ross J.M., Sinclair D.A. (2022). Mitochondrial and metabolic dysfunction in ageing and age-related diseases. Nat. Rev. Endocrinol..

[17] Liao X., Butow R.A. (1993). RTG1 and RTG2: two yeast genes required for a novel path of communication from mitochondria to the nucleus. Cell.

[18] Liu Z., Butow R.A. (2006). Mitochondrial retrograde signaling. Annu. Rev. Genet..

[19] Hughes A.L., Gottschling D.E. (2012). An early age increase in vacuolar pH limits mitochondrial function and lifespan in yeast. Nature.

[20] Hughes A.L., Hughes C.E., Henderson K.A., Yazvenko N., Gottschling D.E. (2016). Selective sorting and destruction of mitochondrial membrane proteins in aged yeast. eLife.

[21] Schuler M.H., English A.M., Xiao T., Campbell T.J., Shaw J.M., Hughes A.L. (2021). Mitochondrial-derived compartments facilitate cellular adaptation to amino acid stress. Mol. Cell.

[22] Ruan L., Zhou C., Jin E., Kucharavy A., Zhang Y., Wen Z. (2017). Cytosolic proteostasis through importing of misfolded proteins into mitochondria. Nature.

[23] Khoomrung S., Martinez J.L., Tippmann S., Jansa-Ard S., Buffing M.F., Nicastro R. (2015). Expanded metabolite coverage of Saccharomyces cerevisiae extract through improved chloroform/methanol extraction and tert-butyldimethylsilyl derivatization. Anal. Chem. Res..

[24] Montefusco D.J., Newcomb B., Gandy J.L., Brice S.E., Matmati N., Cowart L.A. (2012). Sphingoid bases and the serine catabolic enzyme CHA1 define a novel feedforward/feedback mechanism in the response to serine availability. J. Biol. Chem..

[25] Cowart L.A., Hannun Y.A. (2007). Selective substrate supply in the regulation of yeast de novo sphingolipid synthesis. J. Biol. Chem..

[26] Hill S.M., Hao X., Liu B., Nyström T. (2014). Life-span extension by a metacaspase in the yeast Saccharomyces cerevisiae. Science.

[27] Holmberg S., Schjerling P. (1996). Cha4p of Saccharomyces cerevisiae activates transcription via serine/threonine response elements. Genetics.

[28] Mulleder M., Calvani E., Alam M.T., Wang R.K., Eckerstorfer F., Zelezniak A. (2016). Functional metabolomics describes the yeast biosynthetic regulome. Cell.

[29] Comyn S.A., Young B.P., Loewen C.J., Mayor T. (2016). Prefoldin promotes proteasomal degradation of cytosolic proteins with missense mutations by maintaining substrate solubility. Plos Genet..

[30] Schneider K.L., Nystrom T., Widlund P.O. (2018). Studying spatial protein quality control, proteopathies, and aging using different model misfolding proteins in S. cerevisiae. Front. Mol. Neurosci..

[31] Schneider K.L., Ahmadpour D., Keuenhof K.S., Eisele-Bürger A.M., Berglund L.L., Eisele F. (2022). Using reporters of different misfolded proteins reveals differential strategies in processing protein aggregates. J. Biol. Chem..

[32] Andersson R., Eisele-Bürger A.M., Hanzén S., Vielfort K., Öling D., Eisele F. (2021). Differential role of cytosolic Hsp70s in longevity assurance and protein quality control. PLoS. Genet..

[33] Ho C.H., Magtanong L., Barker S.L., Gresham D., Nishimura S., Natarajan P. (2009). A molecular barcoded yeast ORF library enables mode-of-action analysis of bioactive compounds. Nat. Biotechnol..

[34] Kawai R., Fujita K., Iwahashi H., Komatsu Y. (1999). Direct evidence for the intracellular localization of Hsp104 in Saccharomyces cerevisiae by immunoelectron microscopy. Cell Stress Chaperones.

[35] Sathyanarayanan U., Musa M., Bou Dib P., Raimundo N., Milosevic I., Krisko A. (2020). ATP hydrolysis by yeast Hsp104 determines protein aggregate dissolution and size in vivo. Nat. Commun..

[36] Harari A., Zoltsman G., Levin T., Rosenzweig R. (2022). Hsp104 N-terminal domain interaction with substrates plays a regulatory role in protein disaggregation. FEBS J..

[37] Liu B., Larsson L., Caballero A., Hao X., Oling D., Grantham J. (2010). The polarisome is required for segregation and retrograde transport of protein aggregates. Cell.

[38] Jia Y., Rothermel B., Thornton J., Butow R.A. (1997). A basic helix-loop-helix-leucine zipper transcription complex in yeast functions in a signaling pathway from mitochondria to the nucleus. Mol. Cell. Biol..

[39] Sekito T., Thornton J., Butow R.A. (2000). Mitochondria-to-nuclear signaling is regulated by the subcellular localization of the transcription factors Rtg1p and Rtg3p. Mol. Biol. Cell..

[40] Buschhorn B.A., Kostova Z., Medicherla B., Wolf D.H. (2004). A genome-wide screen identifies Yos9p as essential for ER-associated degradation of glycoproteins. FEBS Lett..

[41] Yang M., Vousden K.H. (2016). Serine and one-carbon metabolism in cancer. Nat. Rev. Cancer..

[42] Lee J.C.-Y., Tsoi A., Kornfeld G.D., Dawes I.W. (2013). Cellular responses to l-serine in Saccharomyces cerevisiae: roles of general amino acid control, compartmentalization, and aspartate synthesis. FEMS Yeast. Res..

[43] González A., Hall M.N. (2017). Nutrient sensing and TOR signaling in yeast and mammals. EMBO J.

[44] Kobayashi J., Sasaki D., Hara K.Y., Hasunuma T., Kondo A. (2022). Metabolic engineering of the l-serine biosynthetic pathway improves glutathione production in Saccharomyces cerevisiae. Microb. Cell. Fact..

[45] Borghouts C., Benguria A., Wawryn J., Jazwinski S.M. (2004). Rtg2 protein links metabolism and genome stability in yeast longevity. Genetics.

[46] Kirchman P.A., Kim S., Lai C.Y., Jazwinski S.M. (1999). Interorganelle signaling is a determinant of longevity in Saccharomyces cerevisiae. Genetics.

[47] Rios-Anjos R.M., Camandona V.L., Bleicher L., Ferreira-Junior J.R. (2017). Structural and functional mapping of Rtg2p determinants involved in retrograde signaling and aging of Saccharomyces cerevisiae. PLoS One.

[48] Liu Z., Sekito T., Spırek M., Thornton J., Butow R.A. (2003). Retrograde signaling is regulated by the Dynamic interaction between Rtg2p and Mks1p. Mol. Cell..

[49] Jazwinski S.M. (2013). The retrograde response: when mitochondrial quality control is not enough. Biochim. Biophys. Acta.

[50] Sekito T., Liu Z., Thornton J., Butow R.A. (2002). RTG-dependent mitochondria-to-nucleus signaling is regulated by MKS1 and is linked to formation of yeast prion [URE3]. Mol. Biol. Cell..

[51] Pray-Grant M.G., Schieltz D., McMahon S.J., Wood J.M., Kennedy E.L., Cook R.G. (2002). The novel SLIK histone acetyltransferase complex functions in the yeast retrograde response pathway. Mol. Cell. Biol..

[52] Masser A.E., Kang W., Roy J., Mohanakrishnan Kaimal J., Quintana-Cordero J., Friedländer M.R. (2019). Cytoplasmic protein misfolding titrates Hsp70 to activate nuclear Hsf1. eLife.

[53] Shunxi W., Xiaoxue Y., Guanbin S., Li Y., Junyu J., Wanqian L. (2023). Serine metabolic reprogramming in tumorigenesis, tumor immunity, and clinical treatment. Adv. Nutr..

[54] Buqué A., Galluzzi L., Montrose D.C. (2021). Targeting serine in cancer: is two better than one?. Trends. Cancer..

[55] Tajan M., Hennequart M., Cheung E.C., Zani F., Hock A.K., Legrave N. (2021). Serine synthesis pathway inhibition cooperates with dietary serine and glycine limitation for cancer therapy. Nat. Commun..

[56] May A.I., Prescott M., Ohsumi Y. (2020). Autophagy facilitates adaptation of budding yeast to respiratory growth by recycling serine for one-carbon metabolism. Nat. Commun..

[57] Minton D.R., Nam M., McLaughlin D.J., Shin J., Bayraktar E.C., Alvarez S.W. (2018). Serine catabolism by SHMT2 is required for proper mitochondrial translation initiation and maintenance of formylmethionyl-tRNAs. Mol. Cell..

[58] Ernst D.C., Downs D.M. (2018). Mmf1p couples amino acid metabolism to mitochondrial DNA maintenance in Saccharomyces cerevisiae. mBio.

[59] Giaever G., Nislow C. (2014). The yeast deletion collection: a decade of functional genomics. Genetics.

[60] Gupta R., Kasturi P., Bracher A., Loew C., Zheng M., Villella A. (2011). Firefly luciferase mutants as sensors of proteome stress. Nat. Methods..

[61] Erjavec N., Larsson L., Grantham J., Nystrom T. (2007). Accelerated aging and failure to segregate damaged proteins in Sir2 mutants can be suppressed by overproducing the protein aggregation-remodeling factor Hsp104p. Genes. Dev..

[62] Kushnirov V.V. (2000). Rapid and reliable protein extraction from yeast. Yeast.

[63] Arita N., Sakamoto R., Tani M. (2020). Mitochondrial reactive oxygen species-mediated cytotoxicity of intracellularly accumulated dihydrosphingosine in the yeast Saccharomyces cerevisiae. FEBS J..

