# Supplementary material for: Perturbations in L-serine metabolism regulate protein quality control through the sensor of the retrograde response pathway RTG2 in Saccharomyces cerevisiae
Source: J Biol Chem. 2025 May 31;301(7):110329. doi: 10.1016/j.jbc.2025.110329 (PMC12269516; doi:10.1016/j.jbc.2025.110329)
Supplement: Supplementary Material [file mmc1.docx]

**Supporting Information**

Pertubations in L-serine metabolism regulates protein quality control through sensor of retrograde response pathway *RTG2* in *S.cerevisae*

Kanika Saxena^1,3^, Rebecca Andersson^1,4^, Per O. Widlund^1^, Sakda Khoomrung^2,5,6,7^ Sarah Hanzén^1,8^, Jens Nielsen^2^, Navinder Kumar^2^, Mikael Molin^2^, Thomas Nyström^1^

From the ^1^Department of immunology and microbiology, Institute for Biomedicine, Gothenburg University, Medicinaregatan 7 A, SE-41390 Göteborg

^2^Systems and Synthetic Biology, Department of Life Sciences, Chalmers University of Technology, Kemivägen 10, SE-412 96 Gothenburg, Sweden

**Current affiliations**:

^3^Translational Science and Experimental Medicine, Early Research and Development, Respiratory and Immunology, Biopharmaceuticals R&D, AstraZeneca AB, Mölndal, Sweden

^4^Stem Cell Aging Group, Program for Clinical Translation of Regenerative Medicine in Catalonia (P-CMR[C]), Institut d’Investigació Biomèdica de Bellvitge (IDIBELL), L’Hospitalet de Llobregat, Barcelona, Spain

^5^Siriraj Center of Research Excellence in Metabolomics and Systems Biology (SiCORE-MSB), Faculty of Medicine Siriraj Hospital, Mahidol University, Bangkok 10700, Thailand

^6^Siriraj Metabolomics and Phenomics Center, Faculty of Medicine Siriraj Hospital, Mahidol University, Bangkok 10700, Thailand

^7^Department of Biochemistry, Faculty of Medicine Siriraj Hospital Mahidol University, Bangkok 10700, Thailand

^8^Clinical Strategy and Innovation, Cochlear Bone Anchored Solutions AB, Mölnlycke, Sweden

**Corresponding authors**: [knksaxena@gmail.com](mailto:knksaxena@gmail.com), [thomas.nystrom@cmb.gu.se](mailto:thomas.nystrom@cmb.gu.se)

Figure S1 **Metabolite profiling of young and old cells of BY4741 a)** graph depicting levels of all metabolites that were detected in young, mid-age and old cells identified by untargeted metabolite profiling using GC-MS (two replicates out of three shown, N=2). Peak area of the metabolite was normalised against the internal standard 10mM anthranilic acid (0.1M HCL), log_2_ values for peak area per weight is plotted. b) Principal Component Analysis (PCA) score plot based on metabolomics data for 2 replicates and mean (N=2) of mid-age, old, and young; exploratory only. c) The variable importance in the project (VIP) of metabolites comparing Mid vs. Young and Old vs. Young. Based on 2 replicates and mean (N=2); exploratory only. d) serine concentration (nmoles/ml) in young, mid-age, and old cells compared between Mca1OE strain with BY4741 (N=3). Concentrations were determined using amino acid standard, and analysis was performed using GC-FID.

Figure S2 ***CHA4* deletion impairs proteostasis in young and old cells a)** Representative full-scale images corresponding to main fig 2a **b)** Representative full-scale images corresponding to main fig 2b **c)** Representative full-scale images corresponding to main fig 2c **d)** BY4741 & *cha4Δ* with or without myriocin (0.5µM) were grown at 30^o^C to log phase. Percentage cells with Guk1-7ts-GFP aggregates were quantified (N=3) using imageJ. One-way ANOVA p-value=0.012; unpaired t-test p-value=0.046 BY4741 vs *cha4Δ*.

Figure S3 **Genetic interaction between *CHA4* and *RTG2* a)** Percentage cells with Guk1-7ts-GFP aggregates (%) (N=3), cells were grown at 30^o^C; one-way ANOVA p-value=0.0002; parametric paired t-test p-value=0.047 *BY4741* vs *cha4*Δ, **b)** Full image corresponding to main fig 3b **c)** Cycloheximide chase experiment, *cha4*Δ cells were treated with 40ng/ml cycloheximide to mimic growth retardation of *cha4Δrtg2Δ* and grown at 30^o^C, Percentage cells with Guk1-7ts-GFP aggregates (%) analysed using imageJ software, one-way ANOVA p-value=0.0002; parametric paired t-test (*) p-value 0.02 **d)** Fold change in percentage cells with Guk1-7ts-GFP aggregates in presence or absence of 219nM rapamycin treatment, and growth at 30^o^C. **e)** Spot assay of tenfold serially diluted logarithmic phase cultures on YP plates containing either 2% glucose or 2% acetate**.** Incubated at 30^o^C for 5 days. **f)** Western blot analysis showing CTL* degradation over time following cycloheximide treatment (200µg/ml). CTL* protein levels were quantified using imageJ software and were normalised against tubulin expression. **g)** Western blot analysis and its quantification for Guk1-7ts-GFP protein expression normalised to tubulin expression. **h)** Western blot analysis and quantification for Shm1/2 protein expression normalised to pgk1, for BY4741, *cha4Δ*, & *cha4Δrtg2Δ* (N=3) paired t-test was performed. **i)** Spot assay of tenfold serially diluted logarithmic phase cultures on YP plates containing 2% glucose, with or without 1mM H_2_O_2_**.** Incubated at 30^o^C for 2 days. J) mitoSOX red mean fluorescence intensity normalised to baseline fluorescence of unstained cells at 610nm, calculated for 80-100 cells using imageJ software (N=2).

Figure S4 ***RTG2* and *MKS1* regulate proteostasis in *cha4*Δ through non-canonical interaction a)** Representative full-scale image corresponding to main fig 4b. **b)** Percentage cells with Guk1-7ts-GFP aggregates (%) (N=3), cells were grown at 30^o^C; parametric paired t-test (*) p-value=0.042 BY4741 vs *cha4*Δ **c)** Percentage cells with Guk1-7ts-GFP aggregates (%) (N=4), cells were grown at 30^o^C; parametric paired t-test (**) p-value=0.004 *cha4Δ* vs *cha4*Δ*gcn5Δ.*

Figure S5 ***RTG2* overproduction accelerates aggregate clearance upon heat shock a)** Representative full-scale images corresponding to fig 5a. **b)** BY4741 and RTG2OE strain expressing Pro3-1ts-GFP were heat shocked at 38^0^C for 90min (N=5), & 120 (N=4), Percentage cells with Pro3-1ts-GFP aggregates (%), parametric paired t-test (**) p-value=0.003 (90 min); (**) p-value=0.001 (120 min) **c)** BY4741 and *RTG2*OE strain expressing Guk1-7ts-GFP grown at 30^0^C in synthetic complete medium, Percentage cells with Guk1-7ts-GFP aggregates (%) (N=3), parametric paired t-test (*) p-value=0.047 **d)** BY4741 and *RTG2*OE strain expressing Guk1-7ts-GFP were heat shocked at 38^0^C for indicated time intervals, percentage cells with Guk1-7ts-GFP aggregates (%) counted for 100 cells. For time points 50 & 60min (N=2) **e)** BY4741 and *RTG2*OE strain expressing Pro3-1ts-GFP were heat shocked at 38^0^C for indicated time intervals, percentage cells with Guk1-7ts-GFP aggregates (%) 100 cells were counted per strain per time point **f)** Full image for western blot data shown in main fig 5e. **g)** Hsf-1 activity was measured using Luciferase bioluminescence as explained in the methods, all strains were grown at 30^o^C (N=3); one-way ANOVA p-value=0.0008; parametric paired t-test (*) p-value=0.014 *RTG2OE* vs *BY4741;* (*) p-value=0.017 BY4741 vs *rtg2Δ.* **h)** Replicative lifespan of BY4741, and *RTG2OE,* expressing Guk1-7ts-GFP*;* mean replicative lifespan of each strain is indicated in parentheses. Gehan-Breslow-Wilcoxon test performed (*) p-value=0.003 *BY4741*(23.7) vs *cha4Δrtg2Δ* (21.3). **i)** Spot assay of tenfold serially diluted logarithmic phase cultures on YP plates containing either 2% glucose or 3% glycerol. All images were quantified using imageJ software.

**Materials and Methods**

**Spot assay**

Cells grown at 30^o^C to mid-exponential phase were serially diluted ten-fold (1:10^1^-1:10^5^) and spotted on YP plates containing 2% dextrose or YP plates containing 3% glycerol or 2% potassium acetate. For oxidative stress analysis strains were spotted on YP plates containing 2% dextrose with or without 1mM H_2_O_2_. Plates were incubated at 30^o^C for two days to allow maximum growth. For growth on 2% acetate the plates were incubated at 30^o^C for five days.

**Cycloheximide chase**

Concentration of cycloheximide (40ng/ml) was first determined which leads to similar growth retardation in *cha4Δ* as *cha4Δrtg2Δ. cha4Δ* strain was then grown in presence and absence of cycloheximide in YPD at 30^o^C and mid-exponential cells were imaged and analysed for percentage cells with aggregates. For comparison, *cha4Δrtg2Δ* strain was also grown at 30^o^C in YPD without cycloheximide.

**CTL* degradation for proteosome activity measurement**

Selected strains were transformed with plasmid sp70 expressing CTL-star^1^.  Overnight cultures grown in -Ura medium at 30^o^C, were diluted to OD600 = 0.1 and allowed to grow to OD600= 0.5. Cycloheximide was added to 200ug/mL final from a 10mg/mL stock.  Samples were taken at indicated time points by removing 1 mL and adding to 500uL 3x stop mix (450 mM NaCl, 150 mM NaF, 3 mM NaN3, 30 mM EDTA pH 8.0).  Cells were pelleted, supernatant was removed and whole cell protein extraction was performed by alkaline lysis^2^.

**Heat shock and time course**

Indicated strains were grown in YPD at 30^o^C to mid-exponential phase following which they were transferred to 38^o^C, in a water bath for indicated times. Live cells were imaged immediately following heat shock using fluorescence microscopy.

### **Nano-Luciferase assay for Hsf1-activity**

The activity of the Hsf1-dependent stress response was determined as described previously^3^. The strains used in the study carried either plasmid pAM09 (with the luciferase NanoLuc under the control of the HSE element) or the control plasmid pAM10. The cells were grown in minimal media -URA in a white 96-well plate. For the assay, Nano-Glo substrate (Promega) was diluted 1:100 with the supplied lysis buffer and mixed 1:10 with the cell suspensions in the 96-well plate. Bioluminescence was determined immediately, using a plate reader (Biotek Synergy 2 SL). The bioluminescence values were adjusted to the O.D. of each well and the value from vector control was subtracted from each corresponding strain with the NanoLuc-vector. Each value was then normalized to the control strain in each replicate experiment (set to 1). Statistical analyses were performed in GraphPad Prism® 7.01 (GraphPad Software LLC) and Microsoft® Excel® 2016 (Microsoft Corporation).

### **SDS-Page and Western blot**

SDS-PAGE was performed using 4-12 % Bis-Tris gels (Invitrogen NuPAGE®, Thermo Scientific) in a MOPS buffer (Invitrogen NuPAGE®, Thermo Scientific) according to the manufacturer’s instruction. The gels were blotted onto an Immobilion-P PVDF membrane, 0.45 µm pore size, (MERCK Millipore) in a Tris-glycine-methanol transfer buffer overnight. The following antibodies were used for protein detection anti-Shm1/2 (1:2000, Thermo Scientifc, PA5-43278), anti-tub1 (1:10000, company), anti GFP (1: 2000, 9F9.F9, abcam ab1218), anti-c-myc (1: 2000, 9E10, Thermo Scientific, 13-2500). The primary antibodies were detected using 1:10 000 goat anti-mouse or rabbit IgG (H+L) IRDye® 680LT or 800CW (mouse 680LT: LI-COR Biosciences, mouse 800CW: LI-COR Biosciences, rabbit 680LT: LI-COR Biosciences, rabbit 800CW: LI-COR Biosciences), the blots were scanned using the Odyssey® Near Infra-red imaging system (LI-COR) or the ChemiDoc MP imaging system and the images were quantified using ImageJ software. The levels of housekeeping gene (Tub1 or Pgk1) were used for the normalisation.

**MitoSOX Red staining**

Cells were grown to OD = 0.5 in SD-complete medium. Mitosox red was added to 5µM from a 5mM stock in DMSO (Thermo Fischer Scientific) along with a DMSO control.  Stain was incubated for 15min and washed once with SD-complete medium^4^. The cells were imaged with absorption/emission wavelength set to 396/610nm. Mean fluorescence Intensity at 610nm for 80-100 cells was calculated and the background noise was subtracted using FIJI/imageJ software. Average mean fluorescence intensity for each strain was normalised against the baseline mean fluorescence intensity of the unstained cells.

**Drug treatment**

Secondary cultures were inoculated in presence or absence of 219nM rapamycin dissolved in ethanol or 0.5µM myriocin dissolved in DMSO and grown till O.D. 0.5. Cells were harvested and live cells were imaged using fluorescence microscopy. Percentage cells with Guk1-7ts-GFP were quantified using imageJ.

**Strain construction**

| Strain | Genotype | Reference |
| --- | --- | --- |
| BY4741 | MATa *his3Δ*1 l*eu2Δ*0 *met15*Δ0 *ura3*Δ0. | ^5^ |
| *cha4Δ* | MATa *his3Δ*1 l*eu2Δ*0 *met15*Δ0 *ura3*Δ0 *cha4Δ*::k*an*MX4. | ^5^ |
| *ser3Δ* | MATa *his3Δ*1 l*eu2Δ*0 *met15*Δ0 *ura3*Δ0 *ser3Δ*::k*an*MX4. | ^5^ |
| *rtg2Δ* Guk1-7ts-GFP | MATa *his3Δ*1 l*eu2Δ*0 *met15*Δ0 *ura3*Δ0 *rtg2Δ*::natMX6 Guk1-7ts-GFP::HIS. | This study |
| *rtg3Δ Guk1-7ts-GFP* | MATa *his3Δ*1 l*eu2Δ*0 *met15*Δ0 *ura3*Δ0 *rtg3Δ*::natMX6 Guk1-7ts-GFP::HIS. | This study |
| *mks1Δ Guk1-7ts-GFP* | MATa *his3Δ*1 l*eu2Δ*0 *met15*Δ0 *ura3*Δ0 *mks1Δ*::natMX6 Guk1-7ts-GFP::HIS. | This study |
| *RTG2*OE *Guk1-7ts-GFP* | MATa *his3Δ*1 l*eu2Δ*0 *met15*Δ0 *ura3*Δ0 natMX6:pGPD-*RTG2* Guk1-7ts-GFP::HIS. | This study |
| *cha4Δrtg2Δ Guk1-7ts-GFP* | MATa *his3Δ*1 l*eu2Δ*0 *met15*Δ0 *ura3*Δ0 *cha4Δ*::k*an*MX4 *rtg2Δ*::natMX6 Guk1-7ts-GFP::HIS. | This study |
| *cha4Δrtg3Δ Guk1-7ts-GFP* | MATa *his3Δ*1 l*eu2Δ*0 *met15*Δ0 *ura3*Δ0 *cha4*::k*an*MX4 *rtg3*::natMX6 | This study |
| *cha4Δmks1Δ* Guk1-7ts-GFP::HIS | MATa *his3Δ*1 l*eu2Δ*0 *met15*Δ0 *ura3*Δ0 *cha4Δ*::k*an*MX4 *mks1Δ*::natMX6 Guk1-7ts-GFP::HIS. | This study |
| *cha4Δspt7Δ*  Guk1-7ts-GFP::HIS | MATa *his3Δ*1 l*eu2Δ*0 *met15*Δ0 *ura3*Δ0 *cha4Δ*::k*an*MX4 *spt7Δ*::hphNT1 Guk1-7ts-GFP::HIS | This study |
| *cha4Δgcn5Δ*  *Guk1-7ts-GFP* | MATa *his3Δ*1 l*eu2Δ*0 *met15*Δ0 *ura3*Δ0 *cha4Δ*::k*an*MX4 *gcn5Δ*:: hphNT1 Guk1-7ts-GFP::HIS | This study |
| *mks1Δrtg3Δ*  Guk1-7ts-GFP::HIS | MATa *his3Δ*1 l*eu2Δ*0 *met15*Δ0 *ura3*Δ0 *mks1Δ*:: natMX6 *rtg3Δ*:: hphNT1 Guk1-7ts-GFP::HIS | This study |
| *RTG2*OE *rtg3Δ*  Guk1-7ts-GFP::HIS | MATa *his3Δ*1 l*eu2Δ*0 *met15*Δ0 *ura3*Δ0 natMX6:pGPD-*RTG2 rtg3Δ::* hphNT1 Guk1-7ts-GFP::HIS | This study |
| *RTG2*OE  Pro3-1ts-GFP::HIS | MATa *his3Δ*1 l*eu2Δ*0 *met15*Δ0 *ura3*Δ0 kanMX3:pGPD-*RTG2 Pro3-1t*s-GFP::HIS | This Study |

**References**

1 Buschhorn, B. A., Kostova, Z., Medicherla, B. & Wolf, D. H. A genome-wide screen identifies Yos9p as essential for ER-associated degradation of glycoproteins. *FEBS Lett* **577**, 422-426 (2004). <https://doi.org:10.1016/j.febslet.2004.10.039>

2 Kushnirov, V. V. Rapid and reliable protein extraction from yeast. *Yeast* **16**, 857-860 (2000). <https://doi.org:10.1002/1097-0061(20000630)16:9><857::Aid-yea561>3.0.Co;2-b

3 Masser, A. E. *et al.* Cytoplasmic protein misfolding titrates Hsp70 to activate nuclear Hsf1. *eLife* **8**, e47791 (2019). <https://doi.org:10.7554/eLife.47791>

4 Arita, N., Sakamoto, R. & Tani, M. Mitochondrial reactive oxygen species-mediated cytotoxicity of intracellularly accumulated dihydrosphingosine in the yeast Saccharomyces cerevisiae. *Febs j* **287**, 3427-3448 (2020). <https://doi.org:10.1111/febs.15211>

5 Giaever, G. & Nislow, C. The yeast deletion collection: a decade of functional genomics. *Genetics* **197**, 451-465 (2014). <https://doi.org:10.1534/genetics.114.161620>
