# Supplementary figures and images for: Perturbations in L-serine metabolism regulate protein quality control through the sensor of the retrograde response pathway RTG2 in Saccharomyces cerevisiae
Source: J Biol Chem. 2025 May 31;301(7):110329. doi: 10.1016/j.jbc.2025.110329 (PMC12269516; doi:10.1016/j.jbc.2025.110329)

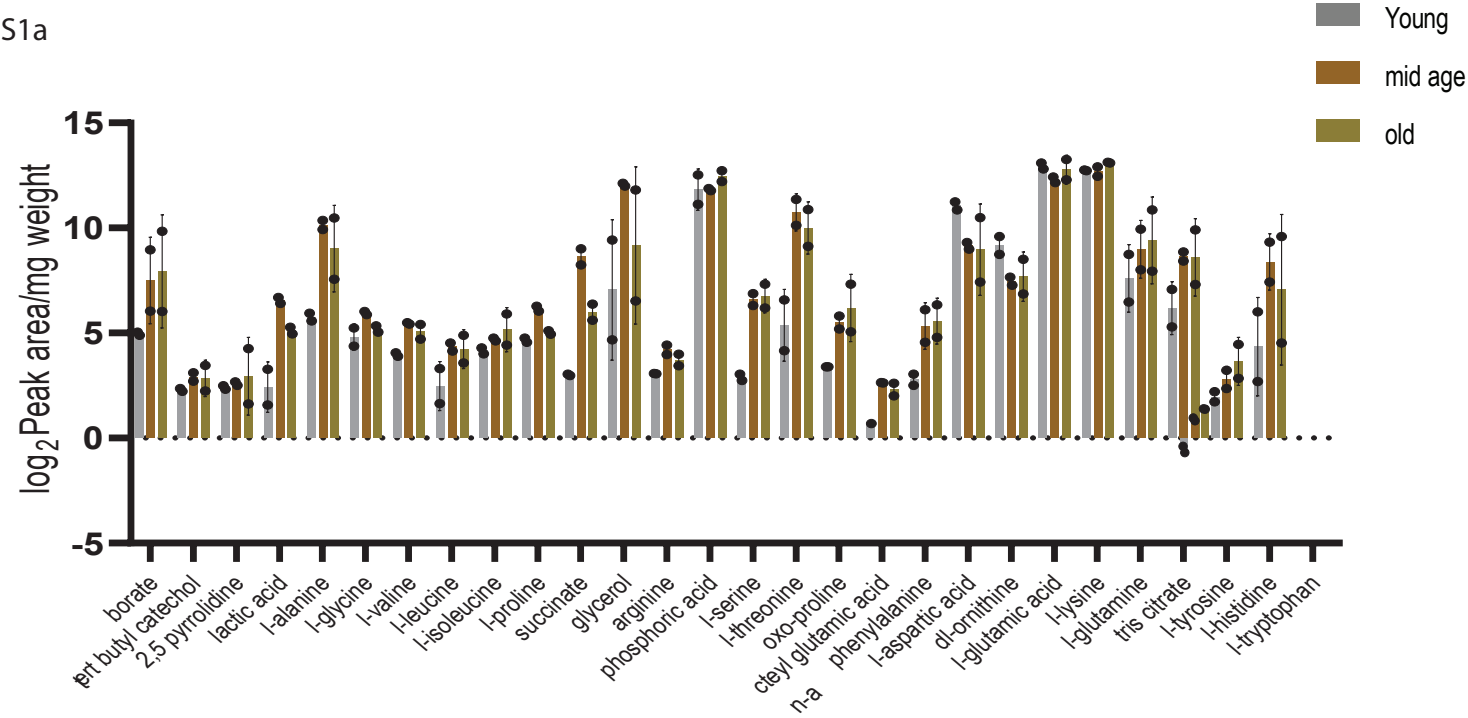

S1b

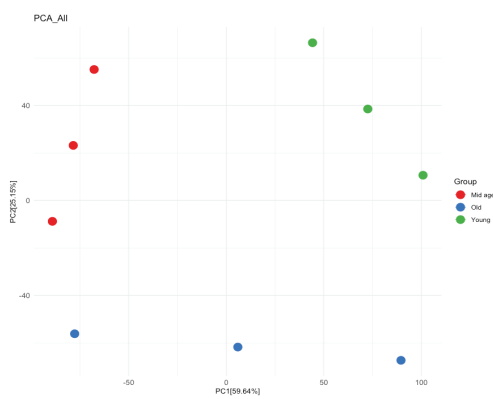

S1c

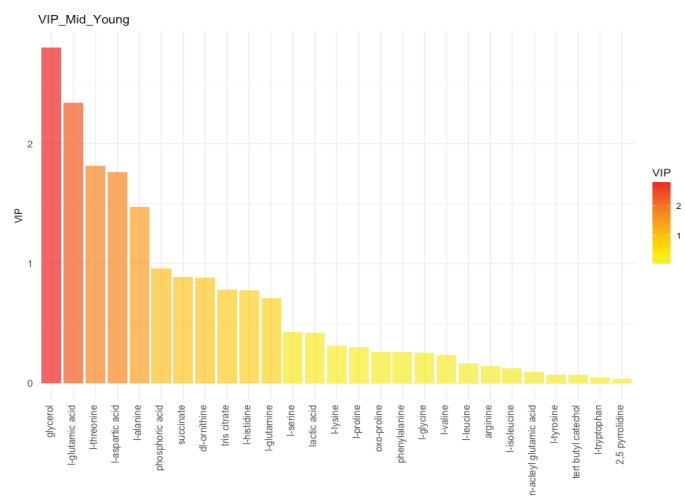

S1d

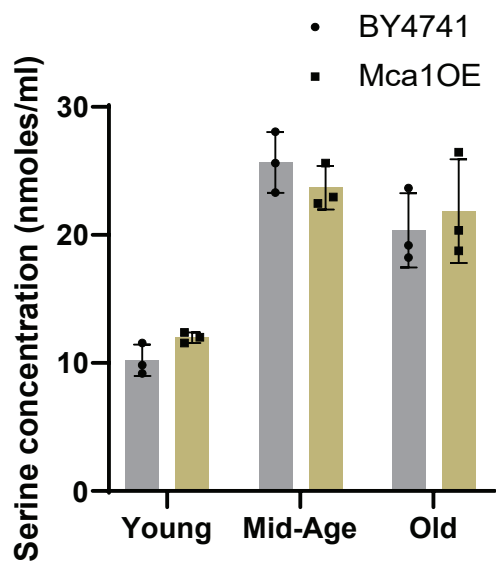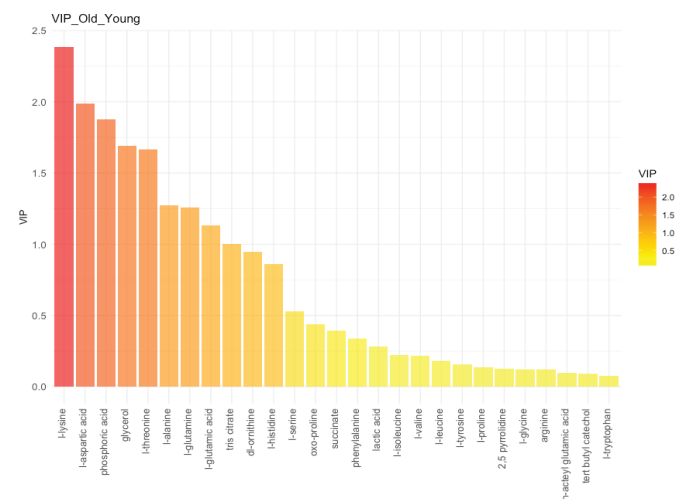

Supplement: Figure S1 [file mmc2.pdf]

S3a

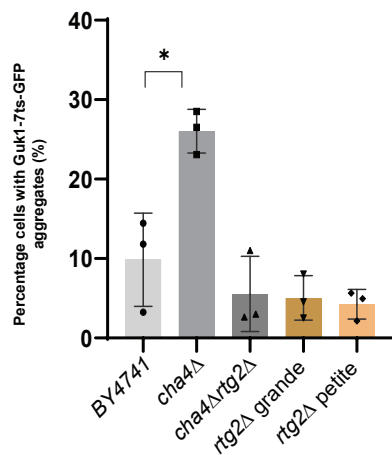

S3c

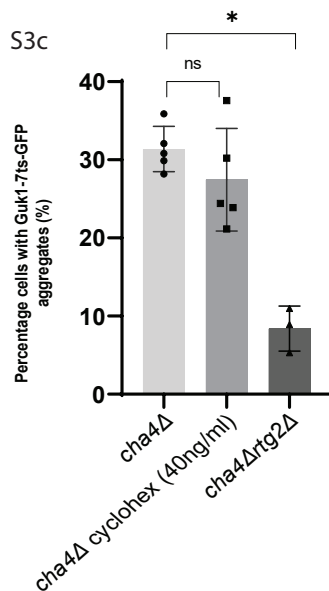

S3d

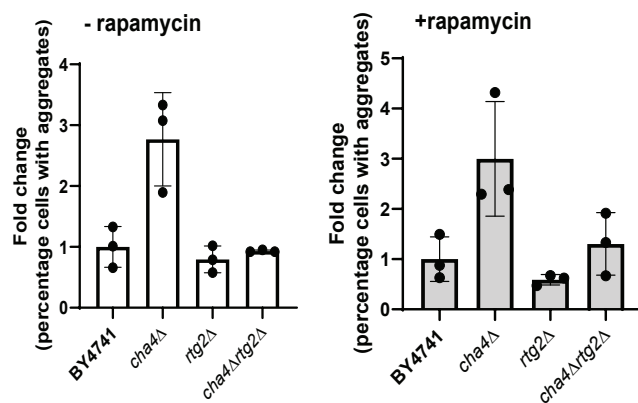

S3b

YP+ 2% Dextrose

YP+ 3% Glycerol

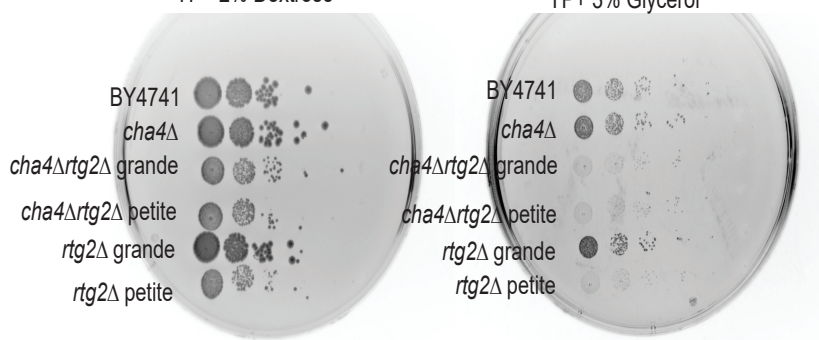

S3e

YP+ 2% Dextrose

YP+ 2% Acetate

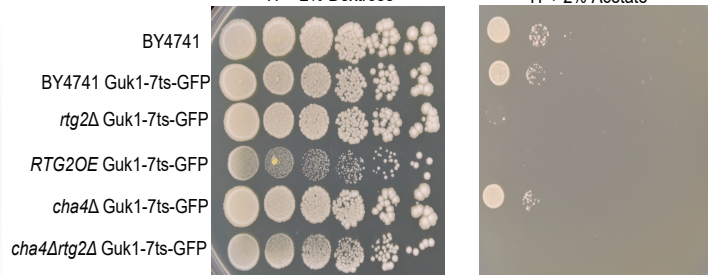

S3f

+cycloheximide

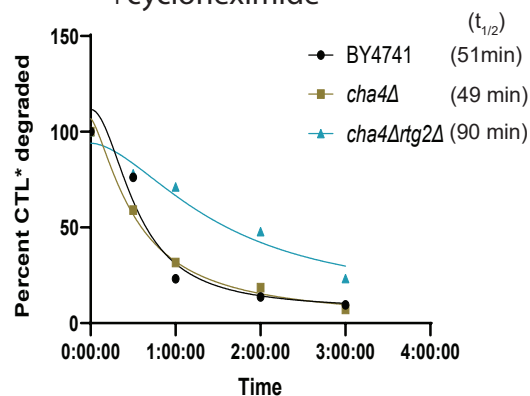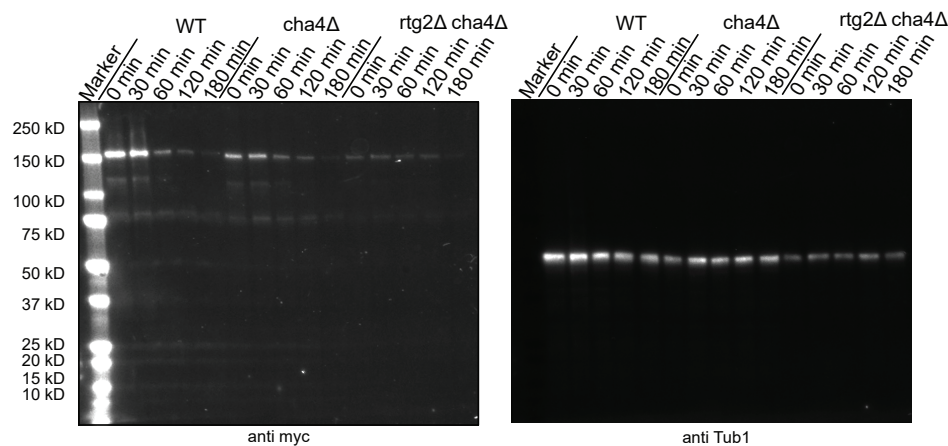

S3g

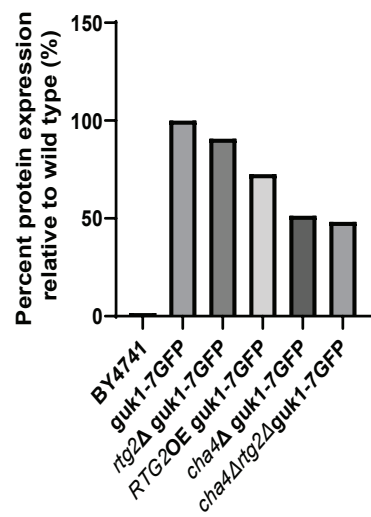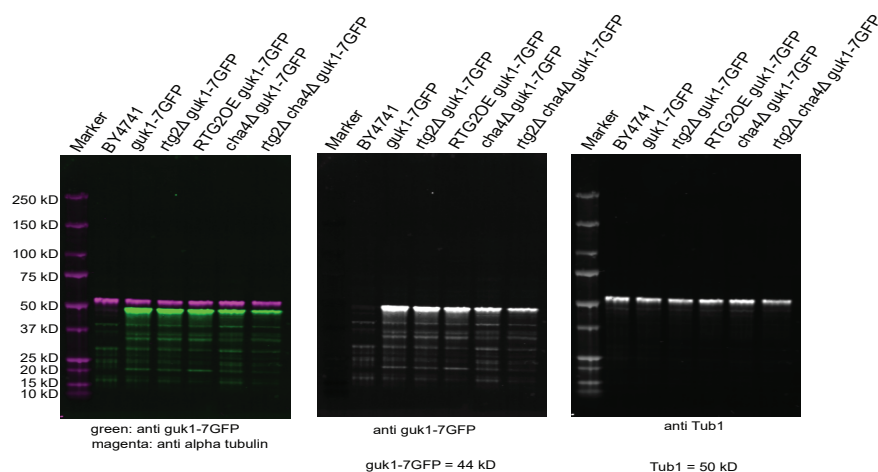

Supplement: Figure S3 part 1 [file mmc4.pdf]

S3h

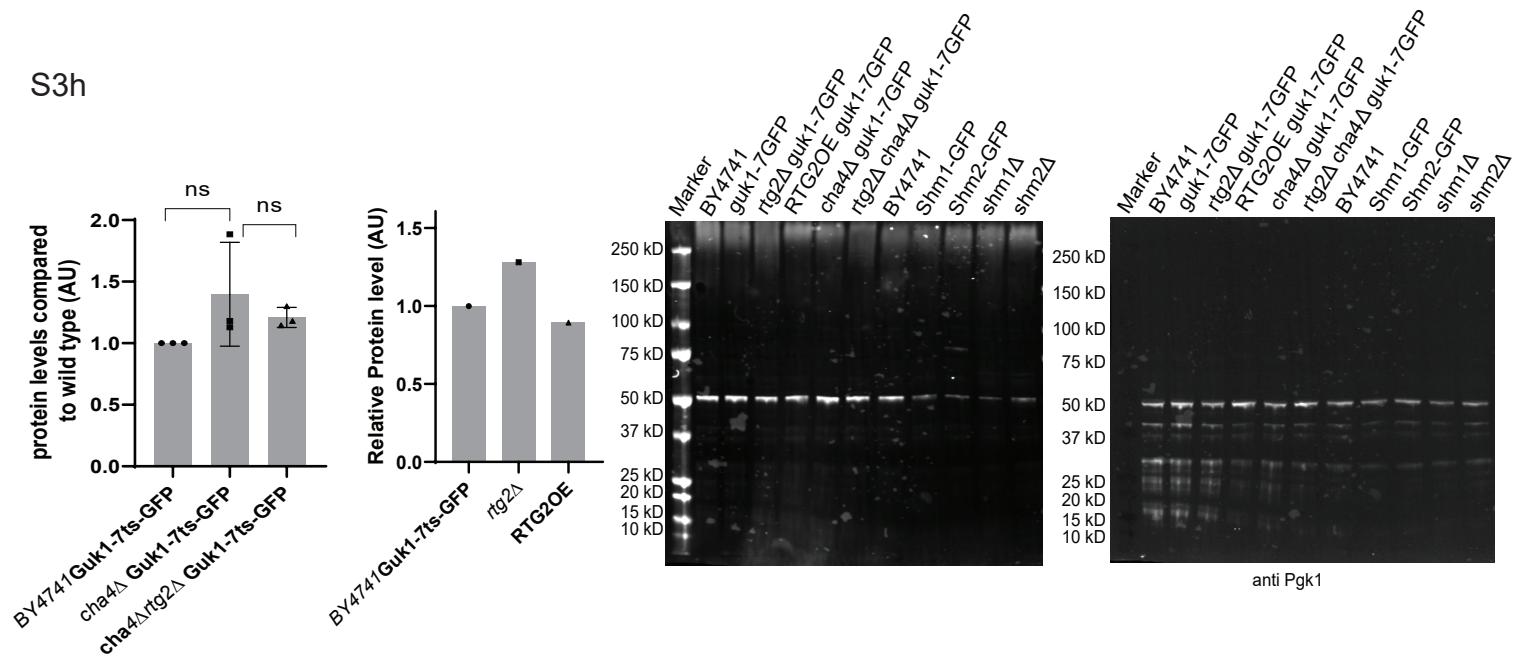

S3i

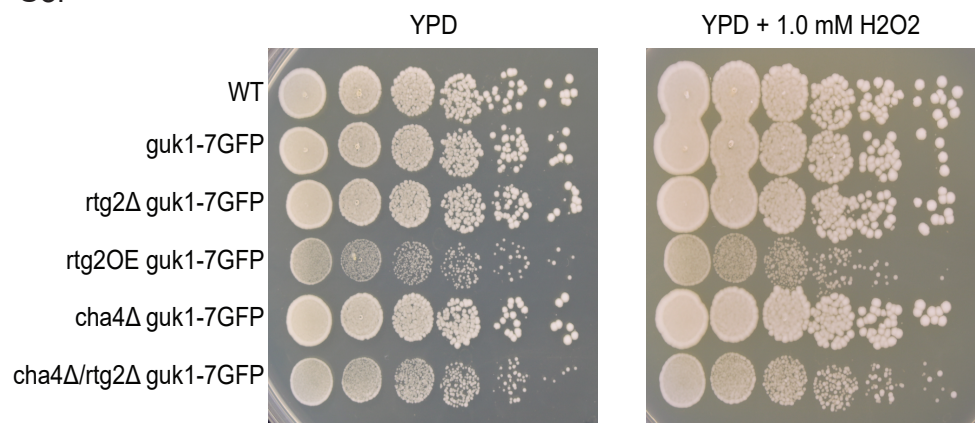

S3j

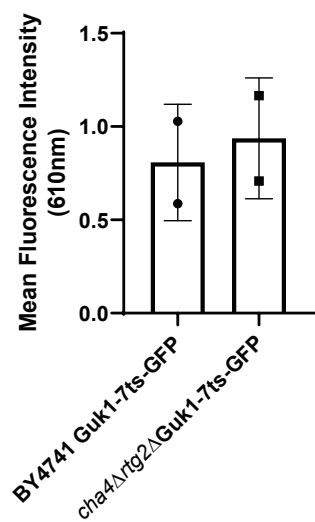

Supplement: Figure S3 part 1 [file mmc5.pdf]

S4a

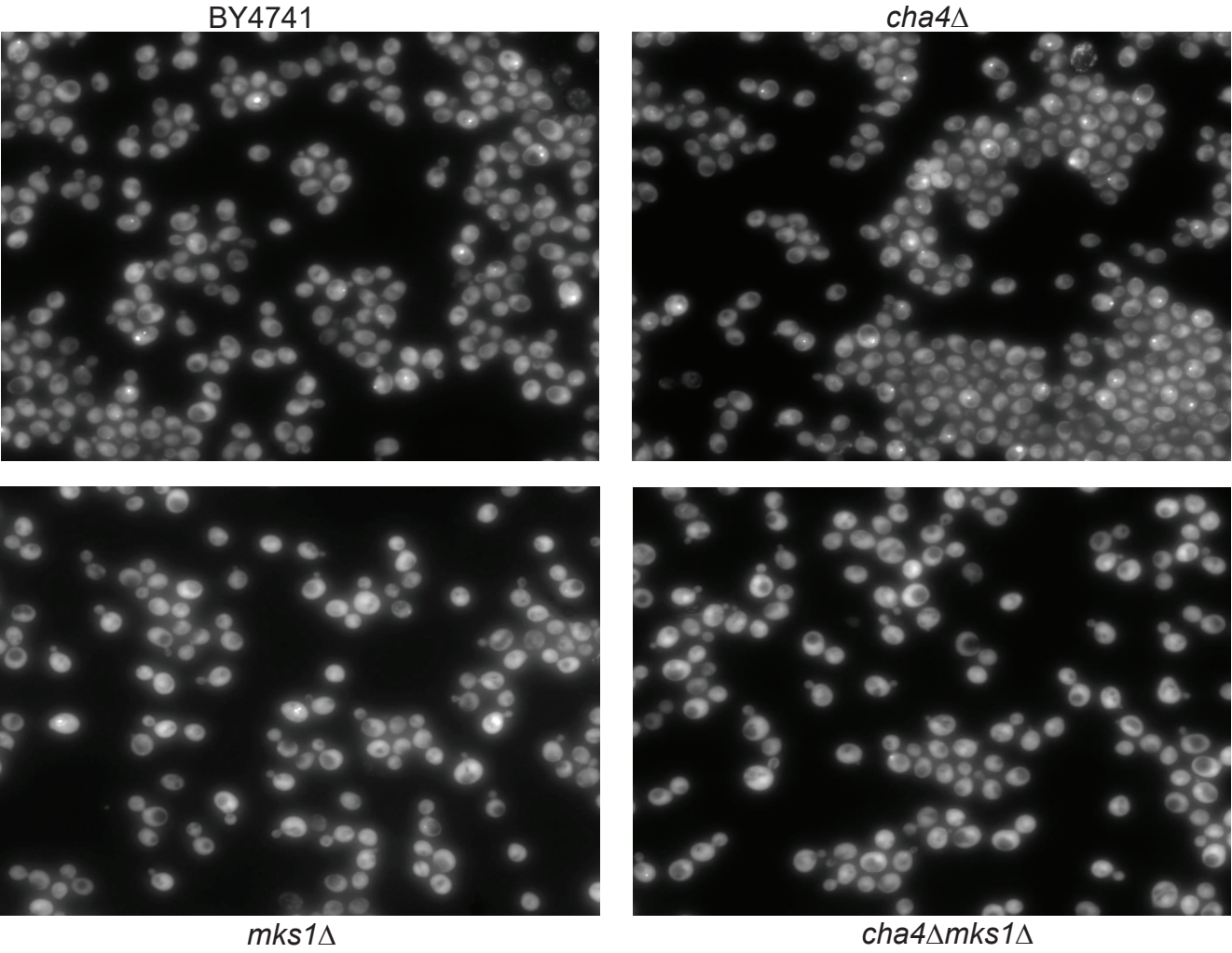

S4b

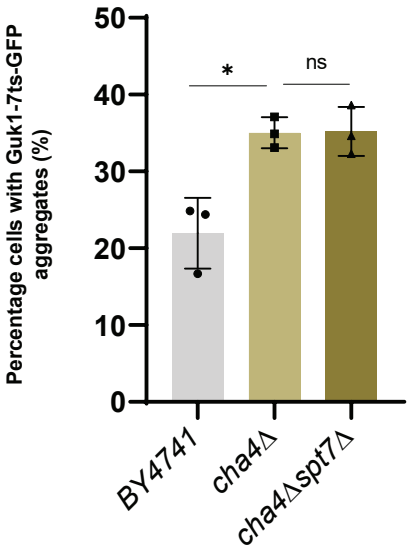

S4c

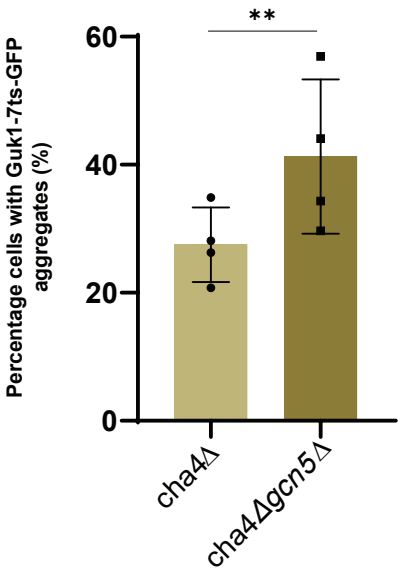

Supplement: Figure S4 [file mmc6.pdf]

S5a

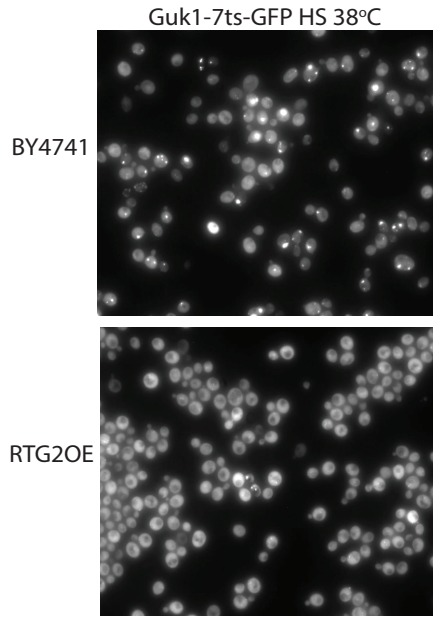

S5b

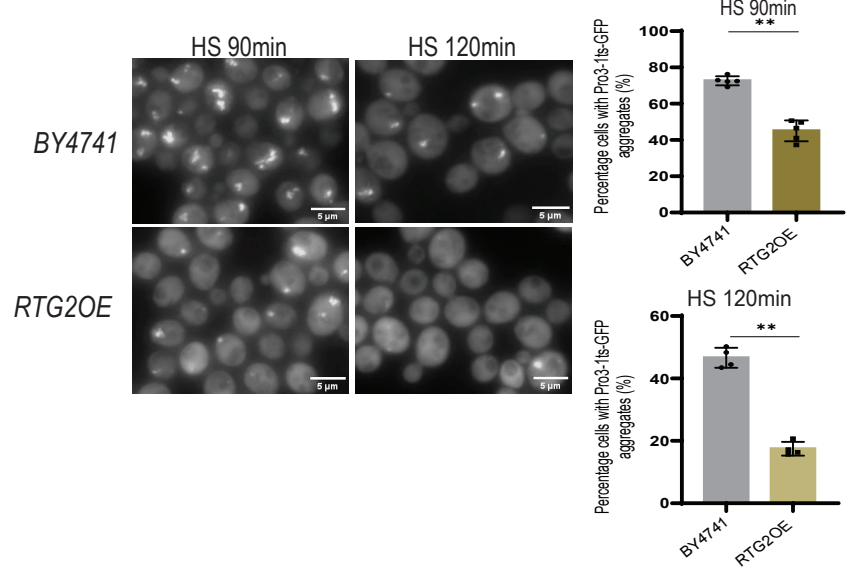

S5c

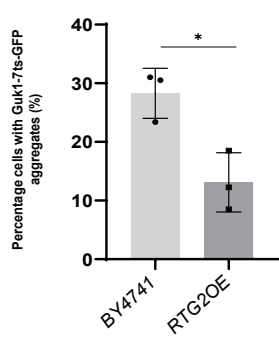

S5d

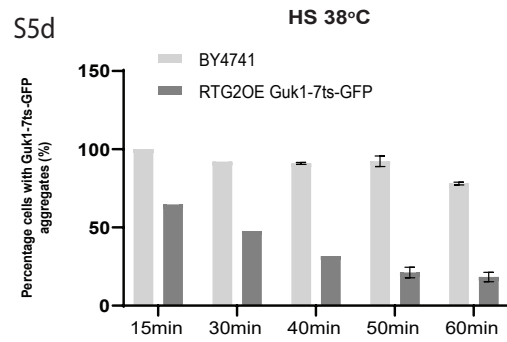

S5f

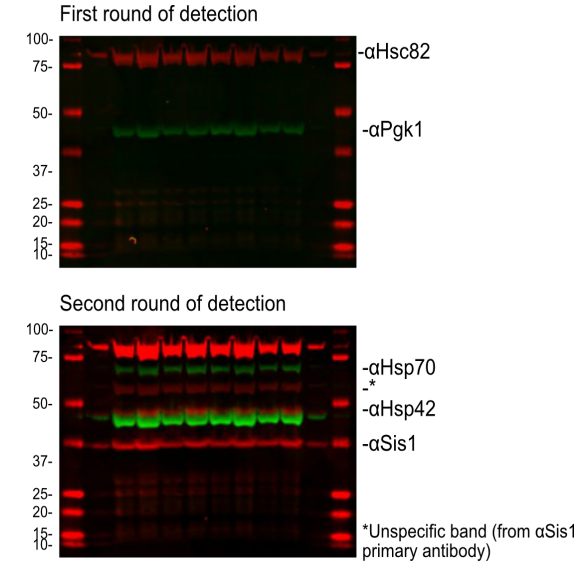

S5e

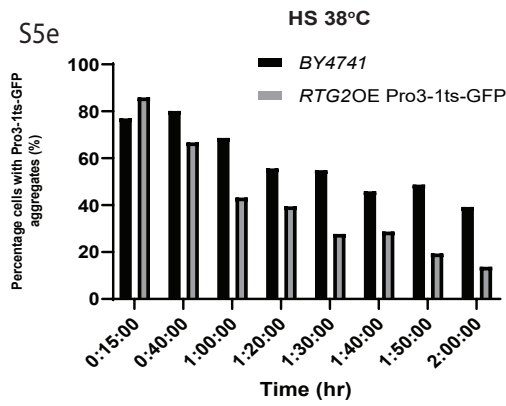

S5g

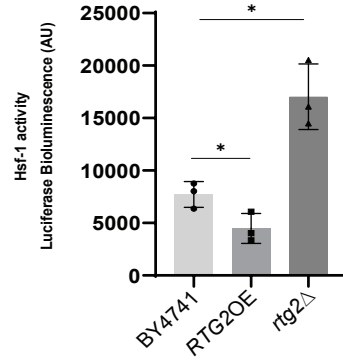

S5h

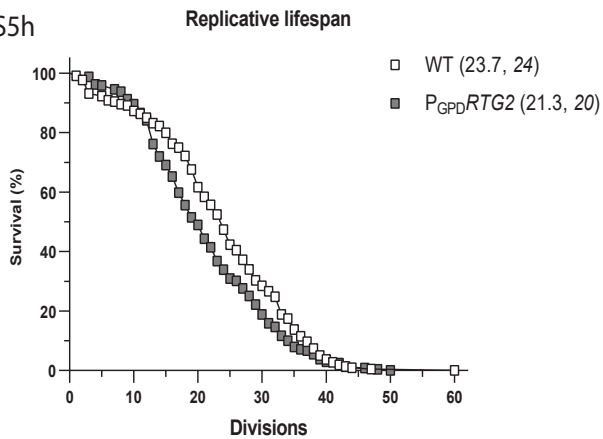

S5i

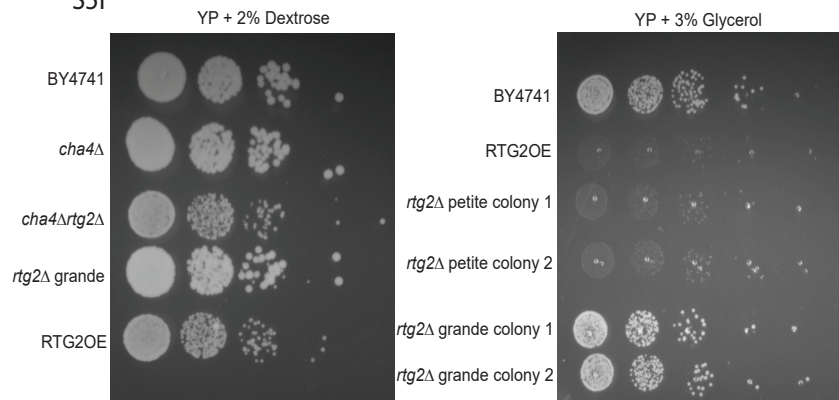

Supplement: Figure S5 [file mmc7.pdf]
