# Supplementary material for: Perturbations in L-serine metabolism regulate protein quality control through the sensor of the retrograde response pathway RTG2 in Saccharomyces cerevisiae
Source: J Biol Chem. 2025 May 31;301(7):110329. doi: 10.1016/j.jbc.2025.110329 (PMC12269516; doi:10.1016/j.jbc.2025.110329)
Supplement: Figure S2 [file mmc3.pdf]

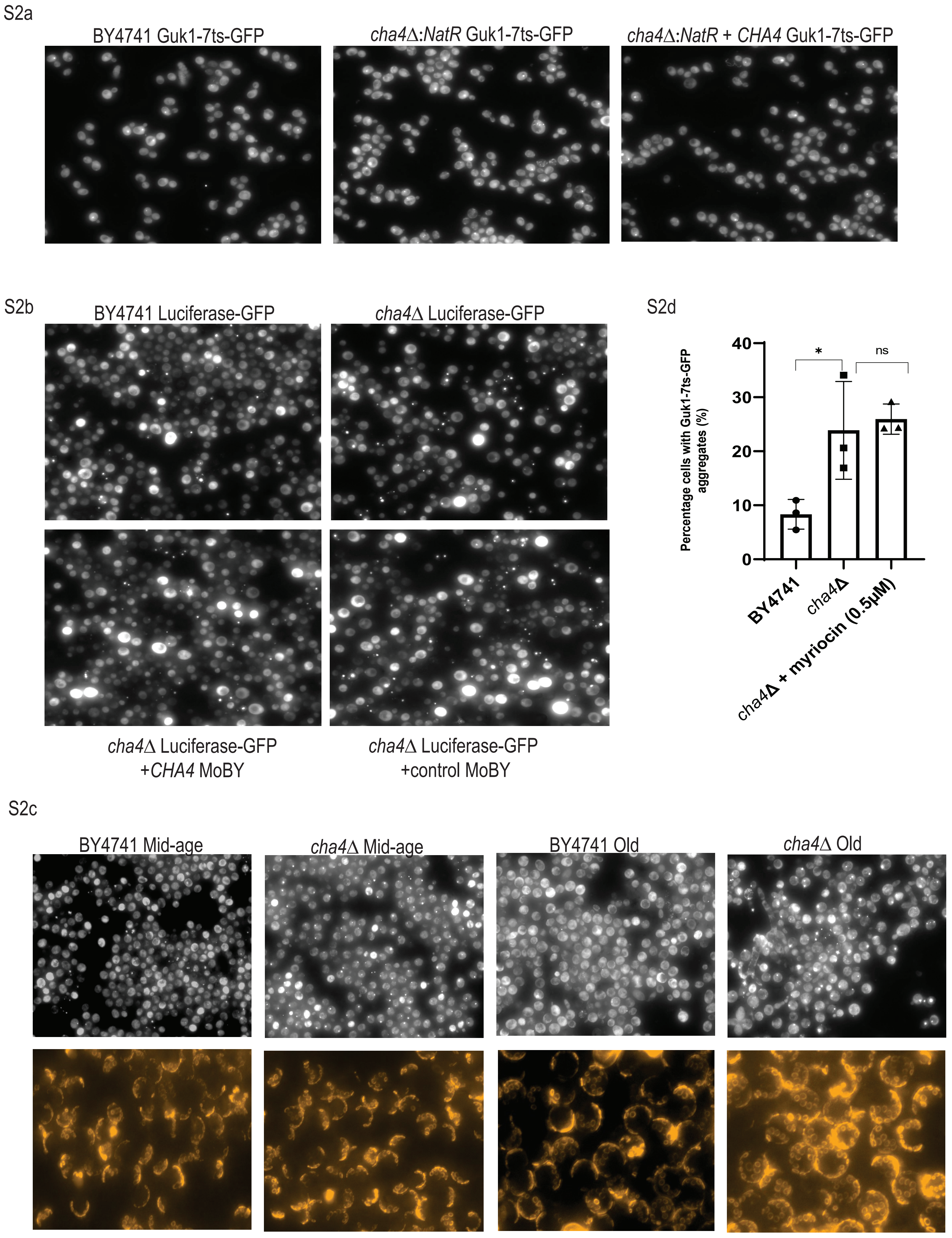
S2a
BY4741 Guk1-7ts-GFP
*cha4*Δ:*NatR* Guk1-7ts-GFP
*cha4*Δ:*NatR* + *CHA4* Guk1-7ts-GFP
S2b
BY4741 Luciferase-GFP
*cha4*Δ Luciferase-GFP
*cha4*Δ Luciferase-GFP +*CHA4* MoBY
*cha4*Δ Luciferase-GFP +control MoBY
S2d
Percentage cells with Guk1-7ts-GFP aggregates (%)
40
30
20
10
0
*
ns
BY4741
*cha4*Δ
*cha4*Δ + myriocin (0.5µM)
S2c
BY4741 Mid-age
*cha4*Δ Mid-age
BY4741 Old
*cha4*Δ Old
